# Antibacterial potential of epiphytic fungi obtained from brown algae of Kenyan coastal waters

**DOI:** 10.1371/journal.pone.0346865

**Published:** 2026-04-09

**Authors:** Aragaw Zemene Sendekie, Kimang’a Andrew Nyerere, Purity Kinya Kaaria

**Affiliations:** 1 Department of Molecular Biology and Biotechnology, Pan African University Institute for Basic Sciences, Technology and Innovation (PAUSTI), Nairobi, Kenya; 2 Department of Medical Biotechnology, Institute of Biotechnology (IoB), University of Gondar, Gondar, Ethiopia; 3 Department of Medical Microbiology, College of Health Sciences, Jomo Kenyatta University of Agriculture and Technology (JKUAT), Nairobi, Kenya; 4 Department of Botany, School of Biological Sciences, Jomo Kenyatta University of Agriculture and Technology (JKUAT), Nairobi, Kenya; Universidad Autonoma de Chihuahua, MEXICO

## Abstract

The rapid rise of antimicrobial resistance, coupled with the limited development of new therapeutic agents, is a critical public health concern that needs urgent and innovative interventions. Marine fungi represent an important but still largely unexplored source of novel and diverse secondary metabolites with potential to combat multidrug-resistant pathogens. The objective of the present study was to investigate the antibacterial properties of fungal epiphytes associated with coastal brown algae against multidrug-resistant pathogens. Epiphytic fungi were isolated from brown algae of the Kenyan coast and identified using morphological and ITS-based molecular approaches. Phylogenetic analysis confirmed the taxonomic placement of the isolates in genera well known for bioactive metabolite production, in agreement with morphological and cultural features. Following the primary screening, nine potent and broadly active isolates were characterized and evaluated for antibacterial activity against multidrug-resistant ESKAPE microorganisms (*Enterobacter cloacae, Acinetobacter baumannii, Enterococcus faecium, Staphylococcus aureus, Klebsiella pneumoniae,* and *Pseudomonas aeruginosa*). The best-performing extracts were subjected to SEM observation, followed by chemical profiling using GC-MS analysis. Both the methanolic and ethyl acetate extracts inhibited bacterial growth, with zones of inhibition ranging from 14 to 30.40 mm. MIC/MBC assays presented potent activity, with values as low as 0.039 mg/mL and 0.156 mg/mL, particularly in extracts from *Alternaria* spp*.* (Dib-4), *Curvularia* spp. (Dib-3), and *Penicillium* spp. (Sac-12). In contrast, some isolates displayed only moderate or selective inhibitory activity. Scanning electron microscopy (SEM) analysis demonstrated morphological alterations in bacterial cells, including membrane blebbing, collapse, and lysis, supporting a membrane-targeting mechanism of action. GC-MS analysis revealed the availability of various bioactive metabolites. These findings indicate that brown seaweed-surface associated fungi from the Kenyan coast represent an untapped reservoir of bioactive constituents with bactericidal activity against multidrug-resistant pathogens. The solvent-dependent recovery of such compounds and the strong activity of selected isolates highlight their promise as candidates for the discovery of novel antimicrobial agents in the future.

## Introduction

Antimicrobial resistance (AMR) has become one of the most pressing public health challenges of the 21^st^ century [1]. Recent estimates indicate that in 2019 alone, nearly five million deaths were associated with drug-resistant bacterial infections worldwide, with the heaviest burden in sub-Saharan Africa [2]. Among the most concerning are the ESKAPE pathogens (*Enterococcus faecium, Staphylococcus aureus, Klebsiella pneumoniae, Acinetobacter baumannii, Pseudomonas aeruginosa,* and *Enterobacter* spp.), which have the ability of “escaping” the effects of existing antimicrobials [3]. These pathogens account for a significant proportion of nosocomial and multidrug-resistant infections worldwide, leading to prolonged hospitalizations, high mortality rates, and escalating healthcare costs [4]. In recognition of their global impact, the World Health Organization (WHO) designated ESKAPE organisms as a top priority for new antibacterial discovery. This has intensified efforts to explore unconventional environments such as marine ecosystems for bioactive metabolites with antibacterial potential.

Encouraged by the idea of “Drugs from the Sea,” the chemists have identified lots of bioactive compounds with novel structures from the rich marine bio-resource in the past fifty years [5]. Among them, marine-derived fungi have contributed an important proportion. Many marine fungal strains were isolated, screened, and reported to produce novel antimicrobial compounds belonging to the alkaloids, macrolides, terpenoids, peptide derivatives, and other structure types [6]. These marine fungal-derived compounds have provided us with new choices to fight infectious diseases. For example, the marine fungus *Pestalotia* sp., isolated from the surface of the brown alga *Rosenvingea* spp., was able to produce a new chlorinated benzophenone compound pestalone, which showed potent antibiotic activity against MRSA, revealing its potential as a new antibiotic. It was also interesting that this fungus produced pestalone only when it was co-cultured with a marine bacterium strain CNJ-328, suggesting that the production of this antibiotic was stimulated by bacterial competition [7]. This example may explain why these marine fungi produce antimicrobial secondary metabolites. These antibiotics may be used as chemical defense weapons against other microorganisms in the marine environment, for the producers themselves, or even for their host [8].

Macroalgae can be divided into three major groups, notably red, green, and brown algae. Brown algae belong to the class Phaeophyceae and contain the pigment fucoxanthin, which gives them a greenish-brown colour. They are used as food in some parts of the world and are habitats for marine organisms [9]. Brown macroalgae (Phaeophyceae) are particularly attractive sampling targets for bio-discovery because their surfaces and mucilage layers create chemically rich microhabitats and because brown algae themselves produce diverse metabolites that shape associated microbial communities [10].

Epiphytic fungi associate with the surface of algae, typically colonizing the outer layers of the thalli without penetrating internal tissues. Their interaction with the host is influenced by various ecological factors, including nutrient exchange and habitat conditions. Given the unique ecological setting of the Kenyan coast, its algicolous fungi may harbor novel chemistry shaped by local host–microbe interactions and environmental pressures [11]. Fungal epiphytes benefit from the organic compounds exuded by seaweeds, such as carbohydrates and phenolic compounds, which serve as nutrient sources. In return, they may contribute to the host’s defense mechanisms, structural integrity, or competitive advantage by producing enormous bioactive substances [12]. Epiphytic fungi associated with brown algae are well known for their ability to biosynthesize a wide range of bioactive metabolites, including phlorotannins, fucoidans, laminarins, sterols, and carotenoids, which possess antibacterial, antifungal, antiviral, anti-inflammatory, antioxidant, and anticancer activities [13]. These metabolites play key ecological roles in defense against herbivory and microbial colonization, and their unique chemical structures have attracted growing interest for pharmaceutical, nutraceutical, and biotechnological applications [14].

Despite these advances, research on algae-associated epiphytic fungi in the Western Indian Ocean (WIO) remains limited [15]. As a less excavated zone, the Kenyan coast probably embodies a rich source of marine derived fungi capable of producing novel antimicrobial compounds. The purpose of this study was therefore to search for antibiotic-producing fungal epiphytes from the local marine habitats. By investigating fungi from this poorly explored marine niche, the study aims to contribute towards the discovery of bioactive metabolites and to scientifically support the pharmaceutical potential of marine-derived fungal resources from the region. This paper reports the isolation, identification, bioassay, and GC-MS-based chemical profiling of the antimicrobial epiphytic fungal isolates against ESKAPE microorganisms, which are of urgent concern in alleviating antimicrobial resistance.

## Materials and methods

### Description of the study site

The study site; Mkomani (Latitude 4° 4’ S and Longitude 39° 41’ E), along the Kenyan coast has been surveyed for marine algae. The coast experiences two distinct monsoon seasons; the northeast monsoon (NEM) locally referred to as “*kaskazi*” and the southeast monsoon (SEM) locally referred to as “*kusi*”. The SEM runs from May to September while NEM from November to March. In between the NEM and SEM, there is one to two months of transition period characterized by variable and lower winds locally referred to as “*matlai*” [16]. These two seasons experience varying pressure, wind, humidity, rain, radiation, and evaporation, that influence local differences in the physical, chemical and biological oceanographic conditions of coastal waters [17].

### Sample collection and preparation

Prior to field sampling, permission was obtained from the National Commission for Science, Technology and Innovation (NACOSTI) under permit number: 634848 (issued on 22/May/2024). Sampling procedures were adhered to the environmental conservation and national regulations. A total of 10 healthy and mature brown algae sample species were collected from the intertidal zones at spring-low tides of the study site in June 2024. The samples were handpicked and given a quick wash with seawater to remove the foreign debris and sand particles. Each seaweed sample was put in a labeled sterile zip-lock polythene bag containing seawater (to avoid desiccation), placed in a cooler box with ice blocks, and transported to the Kenya Marine and Fisheries Research Institute (KMFRI) laboratory for sorting, processing, and identification (Table 1).

**Table 1:** Brown algae collected from the Kenyan coast and taxonomically identified at KMFRI.

No.	Scientific name	Family	Order	Identification code
1.	*Turbinaria conoides*	Sargassaceae	Fucales	B1/6/20/2024
2.	*Dictyota bartayresiana*	Dictyotaceae	Dictyotales	B2/6/20/2024
3.	*Padina tetrastromatica*	Dictyotaceae	Dictyotales	B3/6/20/2024
4.	*Sargassum cristaefolium*	Sargassaceae	Fucales	B4/6/20/2024
5.	*Sargassum oligocystum*	Sargassaceae	Fucales	B5/6/20/2024
6.	*Padina gymnospora*	Dictyotaceae	Dictyotales	B6/6/20/2024
7.	*Turbinaria decurrens*	Sargassaceae	Fucales	B7/6/20/2024
8.	*Sargassum asperifolium*	Sargassaceae	Fucales	B8/6/20/2024
9.	*Sargassum ilicifolium*	Sargassaceae	Fucales	B9/6/20/2024
10.	*Spatoglossum asperum*	Dictyotaceae	Dictyotales	B10/6/20/2024

Key: B – Brown algae.

### Isolation and preservation of fungal epiphytes

The fresh and healthy seaweed samples were initially washed with sterile seawater two times, then gently shaken with sterile glass beads in flasks at 60 rpm for 10 min on shakers, and finally washed again with sterile seawater two times to remove temporarily and loosely adhering free-living fungi [18]. Subsequently, the treated samples were transferred onto Potato Dextrose Agar (PDA) media supplemented with 1 mg/mL chloramphenicol and rubbed on the media surface to inoculate the epiphytic fungi [19]. The PDA were prepared using filtered and autoclaved seawater to closely simulate the natural marine environment and promote optimal growth of marine-derived fungi. The petri-plates were incubated (aerobically) at a temperature of 28 ± 2 °C for about 7–10 days. Pure cultures of the morphologically different fungi were obtained through repeated sub-culturing on PDA plates, then preserved on both slants and potato dextrose broth (in the presence of 15% glycerol) at 4 °C until further identification studies [20]. For convenience, these fungi were given codes and used subsequently in the manuscript (Table 2).

**Table 2:** List of brown algae collected and their associated epiphytic fungal isolates.

Host brown algae and their given codes	Total no. of isolates per host	No. of active isolates	No. of isolates with broad spectrum activity	No. of isolates active against all test bacteria
*Turbinaria conoides* (*Tuc*)	18	1	1	–
*Dictyota bartayresiana* (*Dib*)	11	3	2	2
*Padina tetrastromatica* (*Pat*)	8	1	–	–
*Sargassum cristaefolium* (*Sac*)	17	1	1	1
*Sargassum oligocystum* (*Sao*)	20	5	1	–
*Padina gymnospora* (*Pag*)	10	–	–	–
*Turbinaria decurrens* (*Tud*)	25	8	2	–
*Sargassum asperifolium* (*Saa*)	18	1	1	–
*Sargassum ilicifolium* (*Sai*)	26	2	–	–
*Spatoglossum asperum* (*Spa*)	13	1	1	–
Total	**166**	**23**	**9**	**3**

Key: Isolate codes were assigned according to the binomial name of their host. (-) indicates no activity.

### Primary screening through agar-plug diffusion technique

The pure cultures of epiphytic fungi were first screened for antibacterial properties by agar-plug diffusion assay [21]. The lawns of six test bacterial pathogens (ESKAPEs: *Enterococcus faecium*, *Staphylococcus aureus*, *Klebsiella pneumoniae*, *Acinetobacter baumannii*, *Pseudomonas aeruginosa*, and *Enterobacter cloacae*) were prepared on Mueller Hinton Agar (MHA) using sterile cotton swabs. A sterile cork-borer was used to obtain agar-plugs (6 mm diameter) of actively growing pure cultures of fungi in PDA not enriched with Chloramphenicol. They were transferred to the surface of the above media seeded with test microorganisms adjusted to a 0.5 McFarland standard (1.5 × 10^8^ colony forming units (CFU/mL)) in triplicate [22]. The plates were then refrigerated at 4 °C overnight to facilitate diffusion of metabolites in the plugs; followed by 16–18 h incubation at 37 °C for the bacterial growth. The potential fungal isolates were selected based on the zone of inhibition observed against the test pathogens for secondary screening [23].

### Macroscopic and microscopic identification of fungal epiphytes

Morphological characterization is one of the oldest and reliable methods for the identification of any microorganisms [24]. Based on their bioactivity, nine (n = 9) selected epiphytic fungi were identified using morphological and microscopic features. The cultural characteristics observed such as color and surface colonies (granular, like flour, mounting, slippery), texture, growth period, reverse color, and other features were properly recorded [25]. For the microscopic identification, the hyphae from the pure cultures were placed on a sterile slide and stained with lactophenol cotton blue solution and observed under light microscope (OPTIKA, Italy) [26]. The mycelia and conidiophores of the isolates were observed and then compared with previous descriptions [27]. The identification process was further supported by the taxonomic keys and descriptions provided in the literature authored by [28], which served as an authoritative resource for accurate characterization.

### Molecular characterization of fungal epiphytes

#### Genomic DNA extraction.

A total of nine epiphytic fungal isolates from the collected species of brown seaweeds were subjected to molecular identification. Approximately 100 mg of fungal mycelia from 7–10 days old fungal pure culture grown on PDA was scraped out from the culture plates using a sterile surgical blade. A Quick-DNA^TM^ fungal/bacterial Miniprep kit (Zymo Research Corp. CA 92614, USA) was used to extract the genomic DNA (gDNA) as described by the manufacturer instructions. The extracted gDNA concentration and purity was determined by NanoDrop spectrophotometer (Thermo Fisher Scientific, USA). The concentration was recorded in ng/µL, and purity of DNA was based on the ratio of the optical density (OD) at the wavelength of 260 nm and 280 nm. The quality of the DNA yielded was determined by running gel electrophoresis in a 1% agarose gel.

#### PCR amplification of the ITS gene and sequencing.

The PCR amplification of the fungal internal transcribed spacer (ITS) rDNA gene region from the gDNA was performed using ITS1 (5′TCC GTA GGT GAA CCT GCG G 3′) and ITS4 (5′ TCC TCC GCT TAT TGA TAT GC 3′) primers [29]. Amplification proceeded in a 34-cycle PCR using a Biometra Trio thermocycler (Analytik Jena, Germany) with initial heating at 94 °C for 10 minutes, followed by 34 cycles of the following thermal cycling conditions: denaturation at 94 °C for 70 seconds, annealing at 56 °C for 60 seconds, polymerization at 68 °C for 60 seconds, and final extension at 68 °C for 5 minutes. The presence of PCR products was confirmed using 1% agarose gel electrophoresis in 1 × TAE buffer at 90 voltages for 1 hour and visualized upon staining with Safe-Red. The PCR products were stored at −20 °C before being shipped for sequencing. Sequencing of the PCR products was performed using a commercial service provider (Macrogen Ltd, Singapore). The resulting gene sequences were submitted to the NCBI GenBank database and accession numbers have been given and deposited.

#### Phylogenetic analysis.

The DNA sequences were trimmed and edited to obtain consensus sequences using BioEdit software version 7.0.5.2. A search for similar sequences using BLASTN was performed at the National Center for Biotechnology Information (NCBI) GenBank: https://www.ncbi.nlm.nih.gov/nucleotide/ [30]. From the GenBank sequence database, the closest nucleotide sequences were retrieved and put onto a FASTA file format that had the other newly obtained sequences from the study. Subsequently, all the sequences were aligned using the ClustalW program (http://www.clustal.org) against the nearest neighbors [31]. A neighbor-joining tree of the aligned sequences was constructed using MEGA11 software [32]. Evolutionary distances were computed using the maximum composite Likelihood method [33]. To obtain statistical support values for the branches, bootstrapping was conducted with 1000 replicates [34]. All sites, including gaps in the sequence alignment, were excluded pairwise in the phylogenetic analysis. Using the resultant neighbor-joining tree, each isolate was assigned to the proper taxonomic group.

### Fermentation and extraction of secondary metabolites

Based on the primary screening of potent antibacterial activity, nine epiphytic isolates were selected for further small-scale production of secondary metabolites. Extraction of the secondary metabolites was carried out according to the [35]. A plug of 7-day old mycelium (6 mm x 6 mm) was inoculated into 250 mL conical flasks containing 100 mL of seawater potato dextrose broth (PDB) and was allowed to grow for three weeks on an orbital shaker at 120 rpm to stimulate growth. After three weeks, the culture broth was separated from the mycelium by vacuum filtration (Whatman® qualitative filter paper, Grade 1; Sigma-Aldrich, USA) and the filtrates were extracted three times with an equal volume of ethyl acetate (EtOAc) in a separating funnel. The mycelium was dissolved in methanol under dark condition for two days and the mycelium was separated by filtration. Similarly, methanolic extracts were also prepared and both types of extracts were evaporated under reduced pressure at 40–45 °C using a rotary evaporator to obtain crude extracts. The crude extract was weighed and re-suspended in dimethyl sulfoxide (DMSO) to make a stock solution of 20 mg/mL [36]. The DMSO extracts were kept at −20 °C to be used for future antibacterial assays.

### Antibacterial activity using the disc diffusion assay

Ethyl acetate and methanolic crude extracts of the seaweeds fungal epiphytes were tested against multidrug-resistant ESKAPE pathogens that include two Gram-positive bacteria (*Enterococcus faecium* and *Staphylococcus aureus*) and four Gram-negative bacteria (*Klebsiella pneumoniae*, *Acinetobacter baumannii*, *Pseudomonas aeruginosa*, and *Enterobacter cloacae*). These bacterial clinical isolates were selected as test microbes based on their resistance profiles, and they have previously shown to be resistant to a variety of antibiotics, including ampicillin, tetracycline, methicillin, amikacin, vancomycin and many other antibiotics [37]. Antimicrobial activities of fungal crude extracts were assessed by the disc diffusion method, as in the previous study [38]. The bacterial suspensions at the mid-log phase in Mueller Hinton Broth (MHB) were diluted to 0.5 McFarland standard (1.5 × 10^8^ colony forming units (CFU/mL)). Subsequently, it was disseminated throughout the surface of Mueller-Hinton agar (MHA) plates utilizing sterile cotton swabs, followed by placing the paper discs (6 mm) on the surfaces. Discs were evenly placed on the surface of the medium ensuring that a distance of 12–15 mm away was maintained from the edge of the plate and away from each other. Then, 20 μL of fungal extracts were added to discs at a concentration of 20 mg/mL. Each petri plate contained test sample (extract), positive control (chloramphenicol), and negative control (DMSO). Plates were sealed with parafilm and incubated at 37 °C for 24 hours. The diameter of the clear zone of inhibition, including the disc (6 mm), was measured in millimeters [39]. To ensure visual consistency, the complete diameter was included in the graphs. The test was performed in triplicate.

### Minimum inhibitory concentration and Minimum bactericidal concentration

Minimum inhibitory concentration (MIC) of the ethyl acetate, methanolic, and the combined crude extracts of fungal epiphytes was conducted against drug-resistant ESKAPE pathogens that includes two Gram-positive bacteria (*Enterococcus faecium* and *Staphylococcus aureus*) and four Gram-negative bacteria (*Klebsiella pneumoniae*, *Acinetobacter baumannii*, *Pseudomonas aeruginosa*, and *Enterobacter cloacae*). A modified broth micro dilution method developed by the National Committee for Clinical Laboratory Standards (NCCLS) was employed to ascertain the MIC as described in a previous study [40]. The crude extracts were dissolved in 1% (v/v) dimethyl sulfoxide (DMSO) and working concentrations made in 2-fold serial dilutions where the highest concentration was 10 mg/mL. The bacterial test organisms were grown on Mueller Hinton broth to a turbidity of 0.5 McFarland standard (1.5 × 10^8^ colony forming units (CFU/mL)). 100 µL of Mueller Hinton broth was dispensed into all wells of the micro titer plate. 100 µL of 2-fold extract solution was pipetted into the wells of column 1. Using the pipette, the extract was mixed by sucking up and down 5–8 times. 100 µL of the mixture was withdrawn from column 1 and added to column 2 and then to column 3. The procedure was repeated up to column 10. Then after, 100 µL was discarded from column 10. The final concentration of the extract in each of microplate well was ranging from 10 mg/ml to 0.039 mg/mL. Then, 100 µL of bacteria was poured into wells from columns 1–11. No bacteria were added into column 12 serving as the blank. Cultures without extract and media only were set as a positive control (indicating bacterial growth), while the medium alone functioned as a negative control to confirm sterility. Following incubation of plates at 37 °C for 18 h, MIC was determined using a resazurin-based colorimetric assay, in which a pink color indicated bacterial turbidity and metabolic activity, whereas a blue color signified the absence of growth (inhibition). Accordingly, the MIC was defined as the minimum amount of extract that did not allow observable turbidity. The MIC was performed in triplicate.

Determination of Minimum bactericidal concentration (MBC) value of fungal ethyl acetate, methanolic, and the combined extract was done afterwards based on the reading of the MIC values. Ten microliters from each well that showed no turbidity were applied to the Mueller Hinton Agar (MHA) plate surface and incubated for 24 h at 37 °C. The MBC was observed and recorded as the lowest concentration of fungal extract that caused the reduction of 99.9% in bacterial growth as compared to the growth control [41].

### Mechanism of action

The combined ethyl acetate–methanol extracts of three epiphytic fungi (Dib-3, Dib-4, and Sac-12) exhibiting activity against all test pathogens, using *S. aureus* (Gram-positive) and *Enterobacter cloacae* (Gram-negative) as representatives, were selected for SEM analysis. The test bacterial cultures at the mid-log phase (approximately 1 x 10^8^ CFU/mL) were treated with an equal volume of extract at a concentration corresponding to 2 × MIC and incubated at 37 °C for 1 h. The bacterial pellets were collected at 4000 × g and washed three times by phosphate buffer saline (1x PBS); the bacterial pellet was then fixed at 4 °C with 2.5% glutaraldehyde in PBS overnight. The treated isolates were serially dehydrated using graded ethanol concentrations (30%, 50%, 70%, 90%, and 100%) with an interval incubation period of 15 min and centrifugation at 4000 × g. Finally, samples were air-dried overnight at room temperature and spread on carbon tape, followed by SEM imaging using Scanning Electron Microscope (JCM-7000 NeoScope™, JEOL, Japan) [42].

### Characterization of bioactive metabolites

#### Gas chromatography-coupled mass spectrometry (GC-MS) analysis.

GC-MS is a highly effective analytical technique that integrates the separation efficiency of gas chromatography (GC) with the identification and quantification ability of mass spectrometry (MS) in order to identify bioactive compounds in a complex mixture. It is widely used to analyze volatile and semi-volatile organic compounds across various sample types. In gas chromatography, a gaseous sample is introduced into a column coated with a stationary phase, where compounds are separated according to their boiling points and interactions with the column. Mass spectrometry, the separated compounds are subsequently ionized, fragmented, analyzed, and identified based on their mass-to-charge ratios (m/z) [43]. A gas chromatography mass spectrometry system (Shimadzu GC–MS QP-2010SE) was used to identify the bioactive compounds in fungal extracts. The fungal extracts were analyzed according to the method of Cheseto et al. (2020) [44] with minor modifications. To each fungal extract sample, 1000 μl of the appropriate solvent—ethyl acetate for ethyl acetate extracts and methanol for methanolic extracts, together with 100 mg of Na_2_SO4 as a drying agent was added, vortexed for a minute, extracted by ultra-sonication in sonication bath (Branson 2510, Danbury, CT, USA) for 10 min, centrifuged at 13,000 rpm for five min at 5 °C. The resultant supernatant filtered by passing through glass wool each and a 1.0 μL aliquot was injected into the GC-MS machine for analysis. The system was outfitted with a low-polarity BPX5 capillary column, measuring (30 m × 0.25 mm × 0.25 μm film thickness). The oven temperature was initially set to begin at 55 °C and maintained constant at this level for 1 min. Thereafter, the temperature was then increased at a rate of 10 °C per min until reaching an isothermal temperature of 280 °C. The final retention times were set at 30 mins. The temperature of the injector was established at 250 °C. The carrier gas utilized was helium, with a flow rate of 1.08 mL/min. The sample, diluted at 1% v/v, was introduced (1 μL) into the GC through the AS3000 autosampler in a split ratio of 10:1. The mass detector was configured at 200 °C (for the ion source) and 250 °C (interface temperature). Electron ionization (EI) mass spectra were acquired at 70 eV in full scan mode across an m/z range of 35–550. All the detected components were quantitatively analyzed based on their relative peak areas. Compound identification in the extract was performed qualitatively using the National Institute of Standards and Technology (NIST) Mass Spectral Library [45].

### Statistical analysis and graphing

All assays were performed in triplicates, and the data were reported as mean ± standard deviation of the mean (SD). OriginPro® (2024b) software and GraphPad Prism version 10 (GraphPad Software Inc., San Diego, CA, USA) were employed for graphing and data processing.

## Results

### Primary screening for potential isolates using Agar plug technique

**Fig 1:**
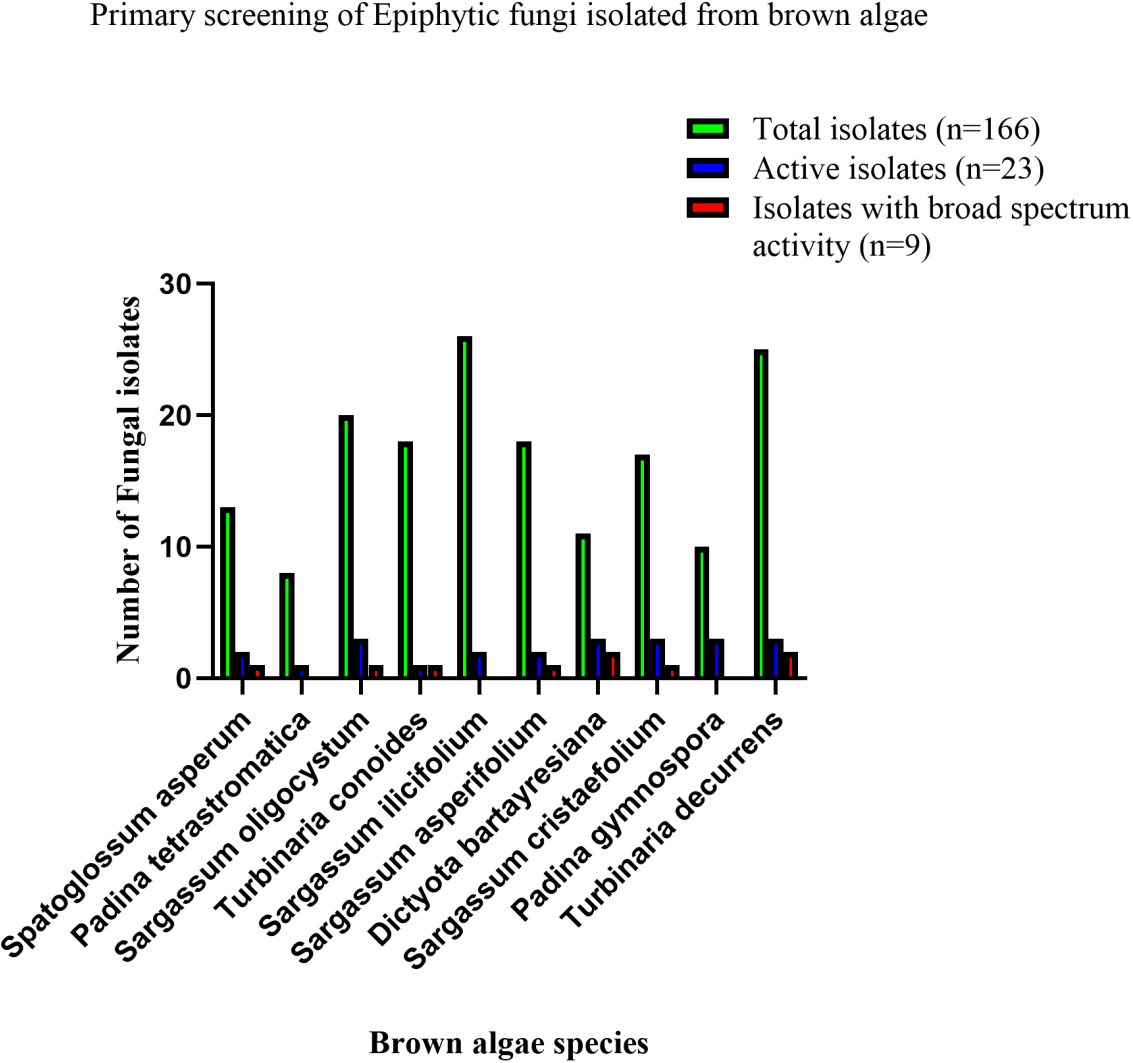
Agar plug-based screening of antibacterial activity in epiphytic fungi from brown algae.

The results showed that the fungal epiphytes isolated in this study exhibiting varying levels of antibacterial activity against the tested bacterial pathogens. Based on the agar plug assay used for primary screening, a total of 166 epiphytic fungal isolates associated with 10 different species of brown algae were assessed for antibacterial activity against drug-resistant ESKAPE microorganisms. Of these, 23 isolates (13.9%) inhibited at least one of the test bacteria, while only nine isolates (5.4%) demonstrated broad-spectrum activity by inhibiting three or more pathogens. These nine morphologically distinct isolates were therefore prioritized for further investigation, as they represented the most promising candidates with wider antibacterial potential. Notably, among these broadly active fungi, three isolates (1.8%) exhibited inhibitory effects against all of the tested pathogens (Fig 1).

### Morphological characterization of fungal epiphytes

The isolated pure cultures of fungal epiphytes were further cultured on petri dishes and left to grow for 7–10 days to get maturation stage. At this stage, the outer structure, colors of the mycelium and spores, as well as the pattern of mycelium formation were observed for morphological identification and assessment. Morphological identification of fungi usually involves both macroscopic and microscopic observations. Macroscopic evaluation involves observing the appearance of the colony, the texture of mycelia, and pigmentation on both sides of the culture plate, whereas microscopic evaluation involves observing the conidia, conidiophore, and branching pattern [46]. Epiphytic fungi isolated from different brown algae showed distinct colony appearance and pigmentation. Based on the morphological characteristics, nine fungal isolates were identified and compared with known species, as demonstrated in Table 3.

**Table 3:** Summary of the cultural and morphological characteristics of fungal epiphytes from brown seaweeds grown on PDA medium after 7-10 days of growth at 28 ± 2 °C.

Code of isolates	Macroscopic characterization			Microscopic characterization		Probable epiphytic fungus
	Color of colony (front/reverse)	Texture	Shape	Mycelium characters	Conidia/Spore Features	
Dib-3	Dark brown to black/ Olivaceous blackish	Velvety, moderately growing	Irregular	Septate, dematiaceous	Conidia curved, 3–4 septa, central cell larger	*Curvularia* sp-2 [49]
Dib-4	Grayish-green to black/ Dark brown	Suede-like, slow-growing	Irregular	Transverse and longitudinal septa, branched, dark-pigmented	Conidia obclavate, dark brown	*Alternaria* sp. [50]
Saa-18	Dark brown to black/Black downy	Suede-like, spreading growth	Circular	Dematiaceous, septate	Crescent-shaped conidia with enlarged central cell, 3-septate	*Curvularia lunata* (sp-1) [47]
Sac-12	Bluish-green/ Yellow to brown	Powdery, compact growth	Circular	Fine, septate mycelium	Small, globose to elliptical conidia in long chains	*Penicillium* sp-2 [20]
Sao-9	Yellow to green/ Brown to yellowish	Granular, compact growth	Irregular	Septate, branched mycelium	Globose to sub-globose conidia	*Talaromyces* sp. [48]
Spa-2	Light brown to black/ Dark brown	Woolly, dense	Circular	Dark-pigmented, septate	Curved conidia, 3-septate, dark median cell	*Curvularia* sp-3 [47]
Tuc-1	White to pale violet/ Reddish	Slightly powdery and ropey texture	Circular	Septate, cottony	Macroconidia with 6–10 septa, slightly curved; Microconidia oval	*Fusarium* sp. [25]
Tud-22	White to black/ Yellowish to brown	Powdery, wooly and spreading	Circular	Compact, hyaline septate	Spherical, rough-walled black conidia	*Aspergillus niger sp-1* [48]
Tud-23	Yellow-green/ Goldish-brown	Powdery, fast-growing and woolly	Circular	Fine, septate, dense and translucent	Round to elliptical conidia in chains	*Aspergillus* sp-2 [20]

**Fig 2:**
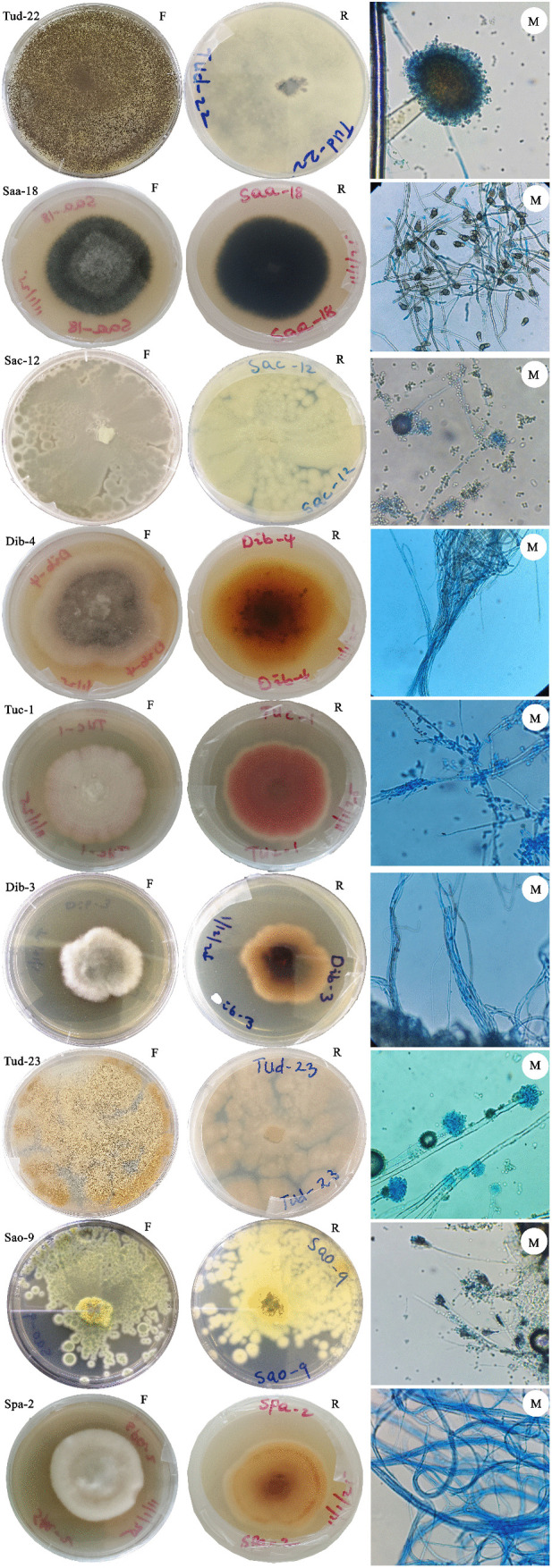
Macroscopic and Microscopic features of the epiphytic fungi grown on PDA medium, incubated at 28 ± 2°C for 7-10 days. Front view **(F)**, Reverse view **(R)**, Microscopic view **(M)**. Magnification of each slide was 40x by observing shape of conidia, and hyphae. The identification process was guided by the taxonomic keys and descriptions provided by Dugan (2008) [28].

As illustrated in Fig 2 and Table 3 below, the isolate corresponding to *Fusarium* sp*.* (Tuc-1) showed white to pale violet colonies with reddish color on the reverse and macroconidia with 6−10 septa [25]. Among the *Alternaria* isolates, *Alternaria* sp. (Dib-4) exhibited grayish-green to black colonies and dark muriform conidia with both transverse and longitudinal septa, featuring a zigzag patterned conidiophore. *Curvularia* sp-1 (Saa-18) had showed crescent-shaped with 3-septate conidia as a hallmark of this species. *Curvularia* sp-2 (Dib-3) exhibited dark brown colonies and geniculate, septate conidiophore, forming curved, multi-septate conidia. *Curvularia* sp-3 (Spa-2) was characterized by light brown, woolly colonies with septate conidiophores bent at the apex and curved conidia [47].

Among the *Aspergillus* isolates, *Aspergillus* sp-1 (Tud-22) produced white-black powdery colonies with smooth-walled conidiophores and rough-walled, spherical conidia, a distinguishing feature of *A. niger*. *Aspergillus* sp-2 (Tud-23) had fast-growing colonies with rough-walled conidiophores and globose conidia arranged in chains. Its reverse side is often a reddish-gold or brown color. Finally, *Talaromyces* sp. (Sao-9*)* was identified based on their biverticillate conidiophores and globose conidia. This isolate (Sao-9) has also displayed yellow-green granular growth [48].

A total of nine (n = 9) epiphytic fungi with a relatively different morphology were identified based on their macroscopic and microscopic features. Each isolate was given a specific code for identification purposes.

### Molecular identification and phylogenetic analysis

**Fig 3:**
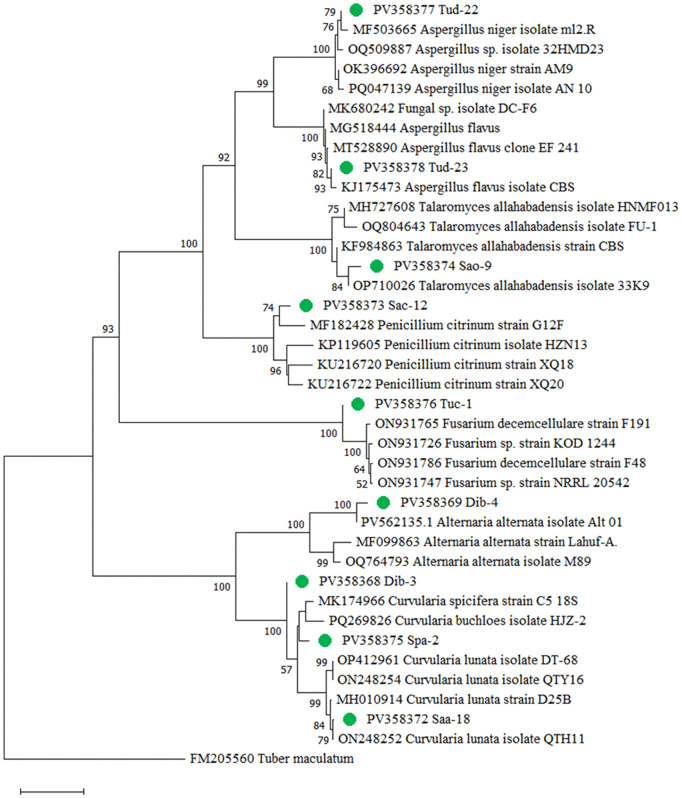
Phylogenetic tree of nine epiphytic fungal isolates showing the genetic relationships among fungal isolates based on ITS sequence data.

The phylogenetic tree reveals distinct clustering of fungal isolates into well-supported clades, corresponding to major genera such as *Aspergillus*, *Penicillium*, *Talaromyces*, *Culvularia*, *Alternaria*, and *Fusarium*. Each isolate clustered with well-supported reference sequences from GenBank, confirming their identities and complementing the morphological observations. Bootstrap support values indicate strong statistical confidence for most branches (Fig 3).

As illustrated in Table 4 and Fig 3, PV358377 (Tud-22) clustered within the *Aspergillus niger* group (bootstrap 79%), showing 100% sequence identity with *A. niger* isolate ml2.R (MF503665). Similarly, PV358378 (Tud-23) grouped within the *A. flavus* lineage, most closely related to isolate CBS (KJ175473) with strong support (bootstrap 93%) and 100% sequence identity. PV358374 (Sao-9) formed a strongly supported cluster (bootstrap 84% and identity 98.73%) within the *Talaromyces allahabadensis* clade, closest to isolate 33K9 (OP710026), with KF984863 (strain CBS) and MH727608 (isolate HNMF013) as additional references. PV358373 (Sac-12) grouped with *Penicillium citrinum*, showing closest similarity to strain G12F (MF182428) with bootstrap 74% and 95.99% identity. PV358376 (Tuc-1) was placed in the *Fusarium decemcellulare* clade (bootstrap 52–100%), with highest affinity to strain F191 (ON931765), supported by ON931766 (strain KOD1244) and ON931786 (strain F48). PV358369 (Dib-4) grouped within the *Alternaria alternata* clade and was closest to the GenBank sequence of isolate A1 (PV562135) with a bootstrap value of 100%. PV358368 (Dib-3) branches adjacent to the strain C5 of *C. spicifera* (MK174966) cluster but joins that clade at a node with low or moderate affinity. This indicates a weak phylogenetic association with *Curvularia spicifera* on the basis of ITS sequence analysis. PV358375 (Spa-2) clustered within *Curvularia*, closest to *Curvularia buchloes* isolate HJZ-2 (PQ269826). Finally, PV 358372 (Saa-18) grouped specifically with *Curvularia lunata*, showing highest similarity to QTH11 (ON248252) with a bootstrap 79% and percent of identity 99.5%. Thus, the isolate Saa-18 was recognized as *Curvularia lunata*, and its morphology matched that of the relevant species. From an overall perspective, the ITS phylogeny resolved the isolates into well-supported clades, and the closest sequence matches were consistent with their cultural and microscopic morphology, providing relatively strong evidence for accurate level of identification.

**Table 4:** BLAST matches for fungal epiphytes isolated from the brown algae of Kenyan coast.

Sample ID	Host seaweed species	Accession Number	Closest taxonomic affiliation from BLAST search	% Query cover	% Identity
Dib-3	*Dictyota bartayresiana*	PV358368	*Curvularia spicifera* MK174966	100	97.90
Dib-4	*Dictyota bartayresiana*	PV358369	*Alternaria alternata* PV562135	99	100.00
Saa-18	*Sargassum asperifolium*	PV358372	*Curvularia lunata* ON248252	100	99.50
Sac-12	*Sargassum cristaefolium*	PV358373	*Penicillium citrinum* MF182428	100	95.99
Sao-9	*Sargassum oligocystum*	PV358374	*Talaromyces allahabadensis* OP710026	100	98.73
Spa-2	*Spatoglossum asperum*	PV358375	*Curvularia buchloes* PQ269826	100	97.64
Tuc-1	*Turbinaria conoides*	PV358376	*Fusarium decemcellulare* ON931765	100	98.77
Tud-22	*Turbinaria decurrens*	PV358377	*Aspergillus niger* MF503665	100	100.00
Tud-23	*Turbinaria decurrens*	PV358378	*Aspergillus flavus* KJ175473	100	100.00

Link for accession numbers: https://www.ncbi.nlm.nih.gov/nuccore/?term=PV358368:PV358378[accn].

BLAST analysis revealed that the sequences of epiphytic fungal isolates showed 95.99–100% sequence similarity with the corresponding sequences in GenBank. To ensure accessibility and future reference, the rDNA-ITS sequences of each isolate were deposited in GenBank database.

### Pairwise genetic distance and phylogenetic tree construction

The number of base differences per site from between sequences are shown in Table 5. This analysis involved nine (n = 9) nucleotide sequences. All ambiguous positions were removed for each sequence pair (pairwise deletion option). Evolutionary analyses were conducted in MEGA11 [33]. The pair-wise genetic distances ranged from 0.002 to 0.526. The maximum pair-wise genetic distance (0.526) was found between Tud-23 (*A. flavus*) and Saa-18 (*C. lunata*). The result can be justified by reason that they both are from different genus. Whereas, the maximum similarity (0.002) was found in between Dib-3 (*C. spicifera*) and Spa-2 (*C. buchloes*). A phylogenetic tree was constructed (Fig 3) from these distance matrix using the software MEGA11 [33].

**Table 5:** Pairwise genetic distances estimated from sequence data of fungal epiphytes from brown algae of the Kenyan coast.

	Dib-3	Dib-4	Saa-18	Sac-12	Sao-9	Spa-2	Tuc-1	Tud-22	Tud-23
Dib-3	–								
Dib-4	0.135	–							
Saa-18	0.046	0.129	–						
Sac-12	0.291	0.298	0.304	–					
Sao-9	0.297	0.306	0.307	0.179	–				
Spa-2	0.002	0.131	0.040	0.297	0.306	–			
Tuc-1	0.303	0.328	0.326	0.241	0.282	0.311	–		
Tud-22	0.496	0.496	0.499	0.436	0.459	0.497	0.487	–	
Tud-23	0.515	0.525	0.526	0.491	0.494	0.517	0.482	0.126	–

### Phylogenetic analysis

In the present study, a phylogenetic tree was constructed using MEGA 11 software to elucidate the evolutionary relationships among the fungal epiphytes isolated. The resulting tree comprised five distinct clades (Fig 3), which represented six different genera: *Aspergillus, Talaromyces, Penicillium, Fusarium*, *Alternaria*, and *Curvularia* all belonging to the phylum Ascomycota. These genera were distributed across three families. The genera *Aspergillus, Talaromyces* and *Penicillium* were from *Trichocomaceae*, *Alternaria* from *Pleosporaceae*, and *Fusarium* from *Nectriaceae*.

Evolutionary analyses were conducted in MEGA11 [33]. The evolutionary history was inferred based on rDNA sequence (ITS1, 5.8S and ITS 4) using the Neighbor-Joining method [51]. The epiphytic fungi isolated in the present study from the marine seaweeds were represented in green circles (Fig 3). The percentage of replicate trees in which the associated taxa clustered together in the bootstrap test (1000 replicates) were shown next to the branches [34]. Branches corresponding to partitions reproduced in less than 50% bootstrap replicates were collapsed. The evolutionary distances were computed using the p-distance method and were in the units of the number of base differences per site. All ambiguous positions were removed for each sequence pair (pairwise deletion option) [51].

### Antibacterial activity using the disc diffusion assay

**Fig 4:**
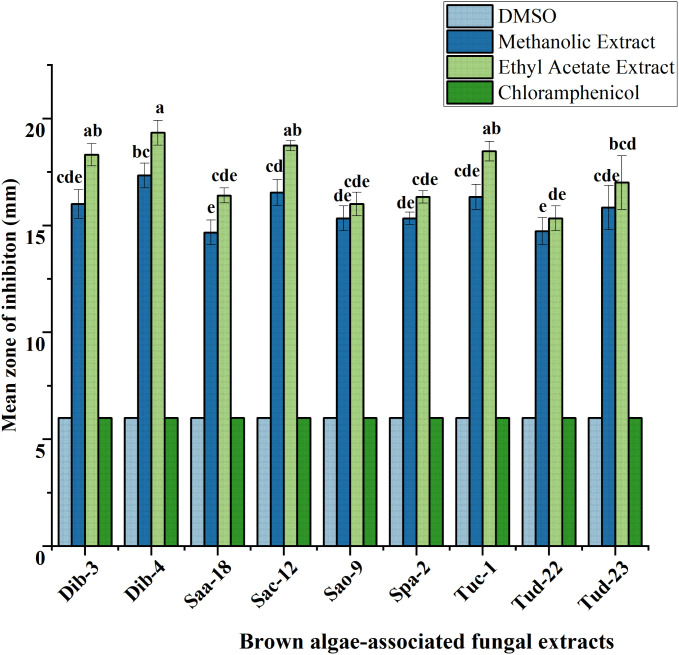
Antibacterial evaluation of Ethyl acetate and Methanol extracts of fungal epiphytes against drug resistant *E. cloacae* by the disc diffusion method. The values are the mean diameter (mm) of triplicate readings (mean ± SD; n = 3). Bars with different letters are significantly different according to Tukey’s post hoc test at p = 0.05. Therefore, any observed difference between them is considered to be statistically significant.

**Fig 5:**
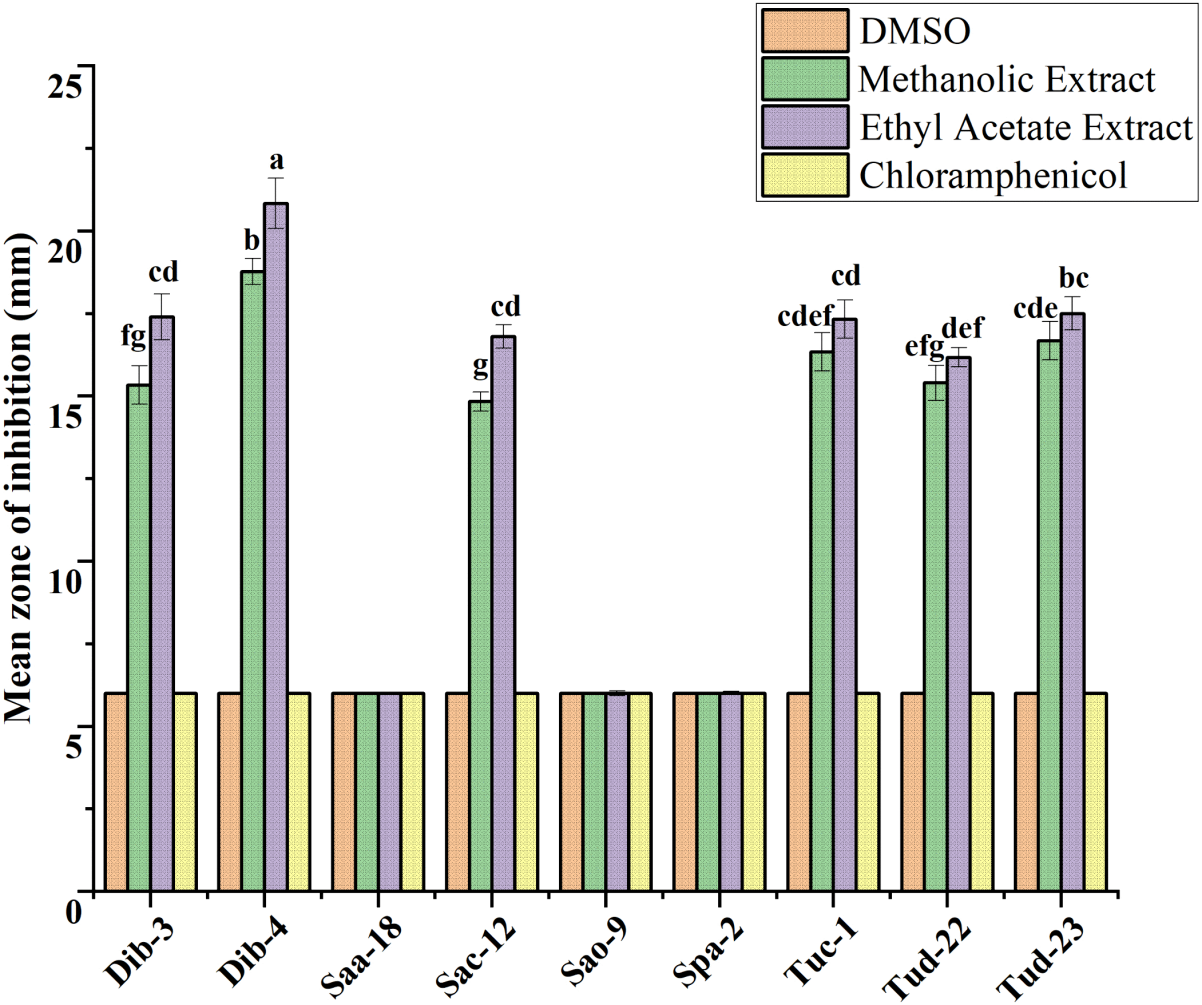
Antibacterial evaluation of Ethyl acetate and Methanol extracts of fungal epiphytes against drug resistant *A. baumannii* by the disc diffusion method. The values are the mean diameter (mm) of triplicate readings (mean ± SD; n = 3). Bars with different letters are significantly different according to Tukey’s post hoc test at p = 0.05. Therefore, any observed difference between them is considered to be statistically significant.

**Fig 6:**
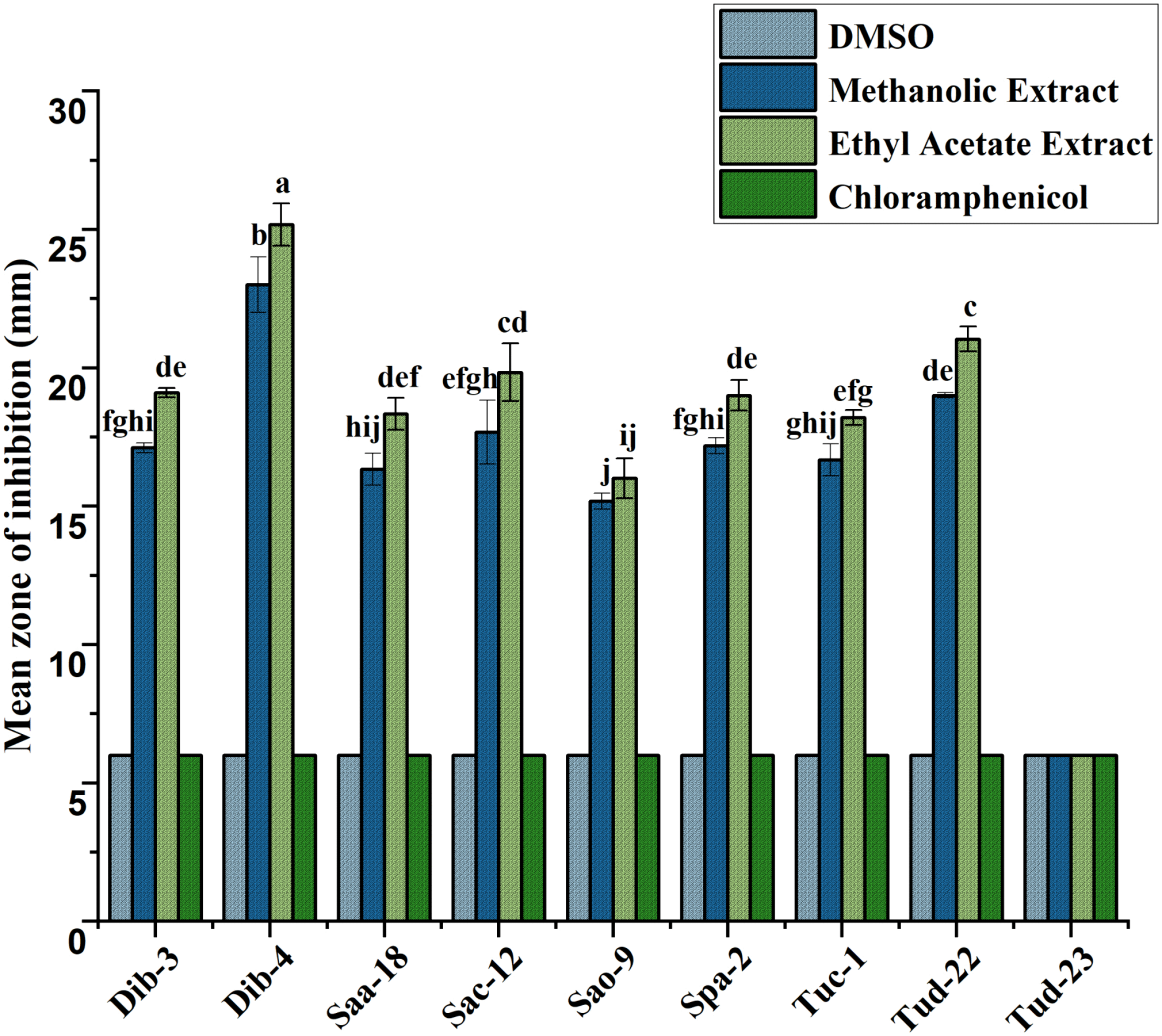
Antibacterial evaluation of Ethyl acetate and Methanol extracts of fungal epiphytes against drug resistant *E. faecium* by the disc diffusion method. The values are the mean diameter (mm) of triplicate readings (mean ± SD; n = 3). Bars with different letters are significantly different according to Tukey’s post hoc test at p = 0.05. Therefore, any observed difference between them is considered to be statistically significant.

**Fig 7:**
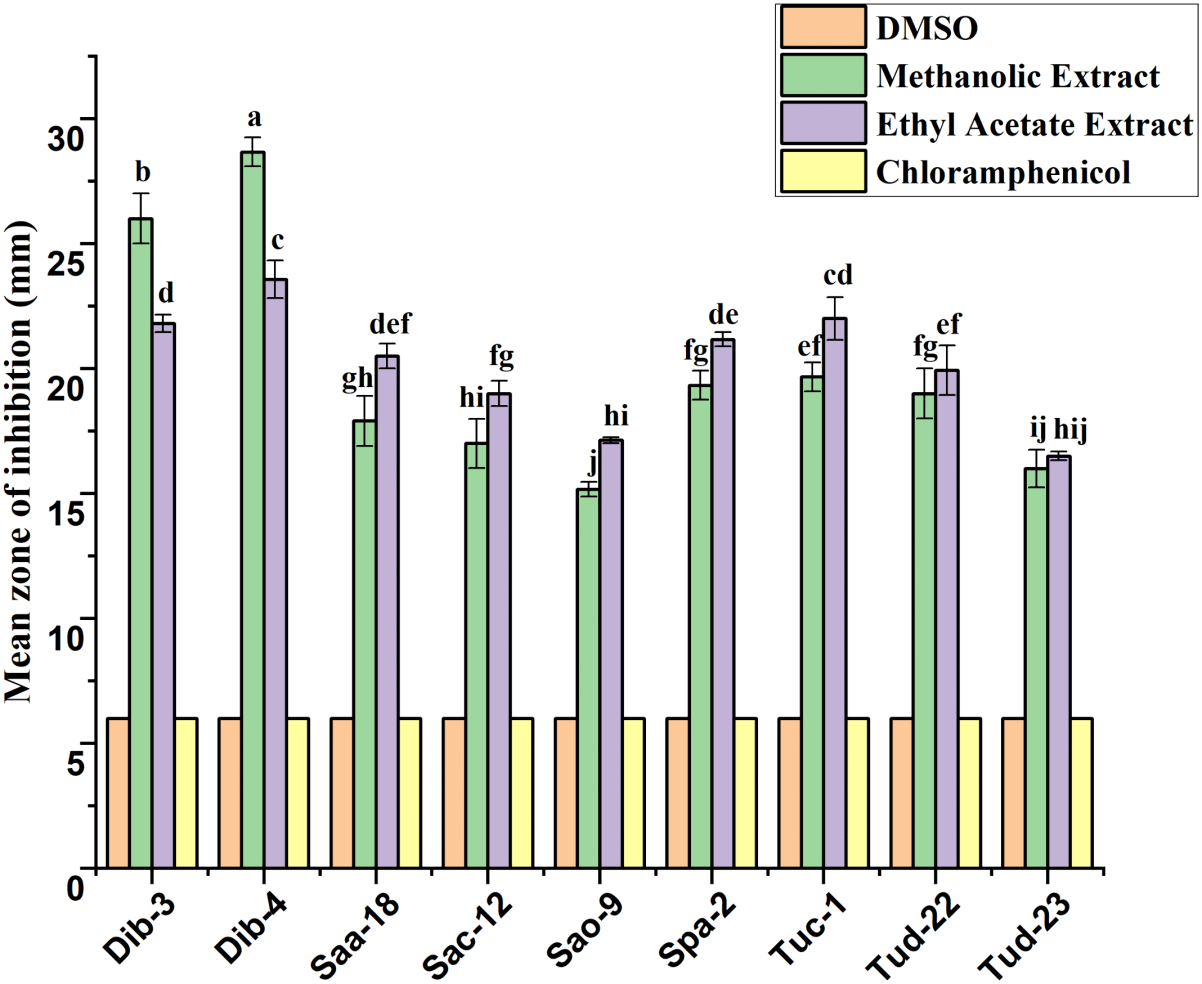
Antibacterial evaluation of Ethyl acetate and Methanol extracts of fungal epiphytes against drug resistant *S. aureus* by the disc diffusion method. The values are the mean diameter (mm) of triplicate readings (mean ± SD; n = 3). Bars with different letters are significantly different according to Tukey’s post hoc test at p = 0.05. Therefore, any observed difference between them is considered to be statistically significant.

**Fig 8:**
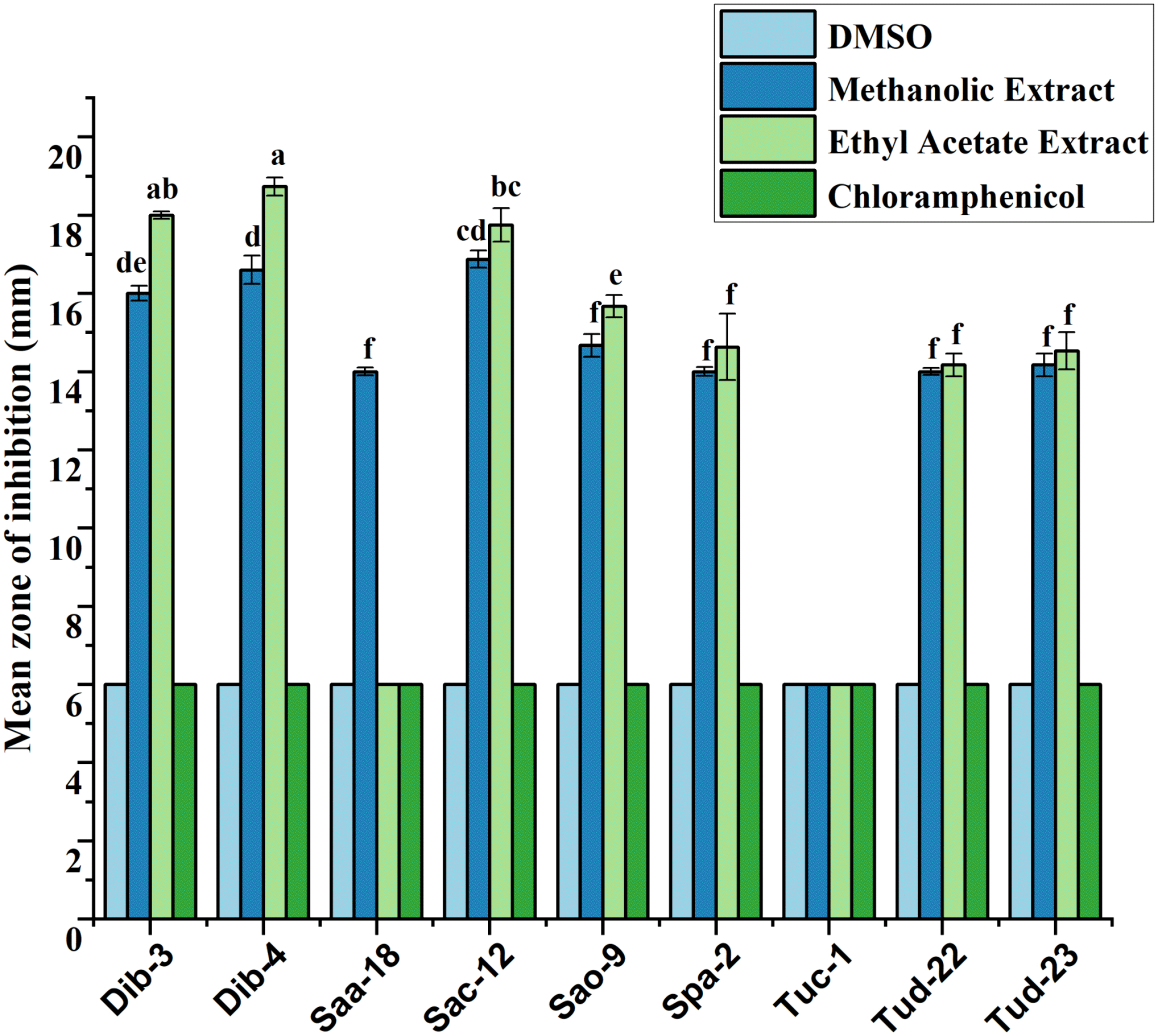
Antibacterial evaluation of Ethyl acetate and Methanol extracts of fungal epiphytes against drug resistant *K. pneumoniae* by the disc diffusion method. The values are the mean diameter (mm) of triplicate readings (mean ± SD; n = 3). Bars with different letters are significantly different according to Tukey’s post hoc test at p = 0.05. Therefore, any observed difference between them is considered to be statistically significant.

**Fig 9:**
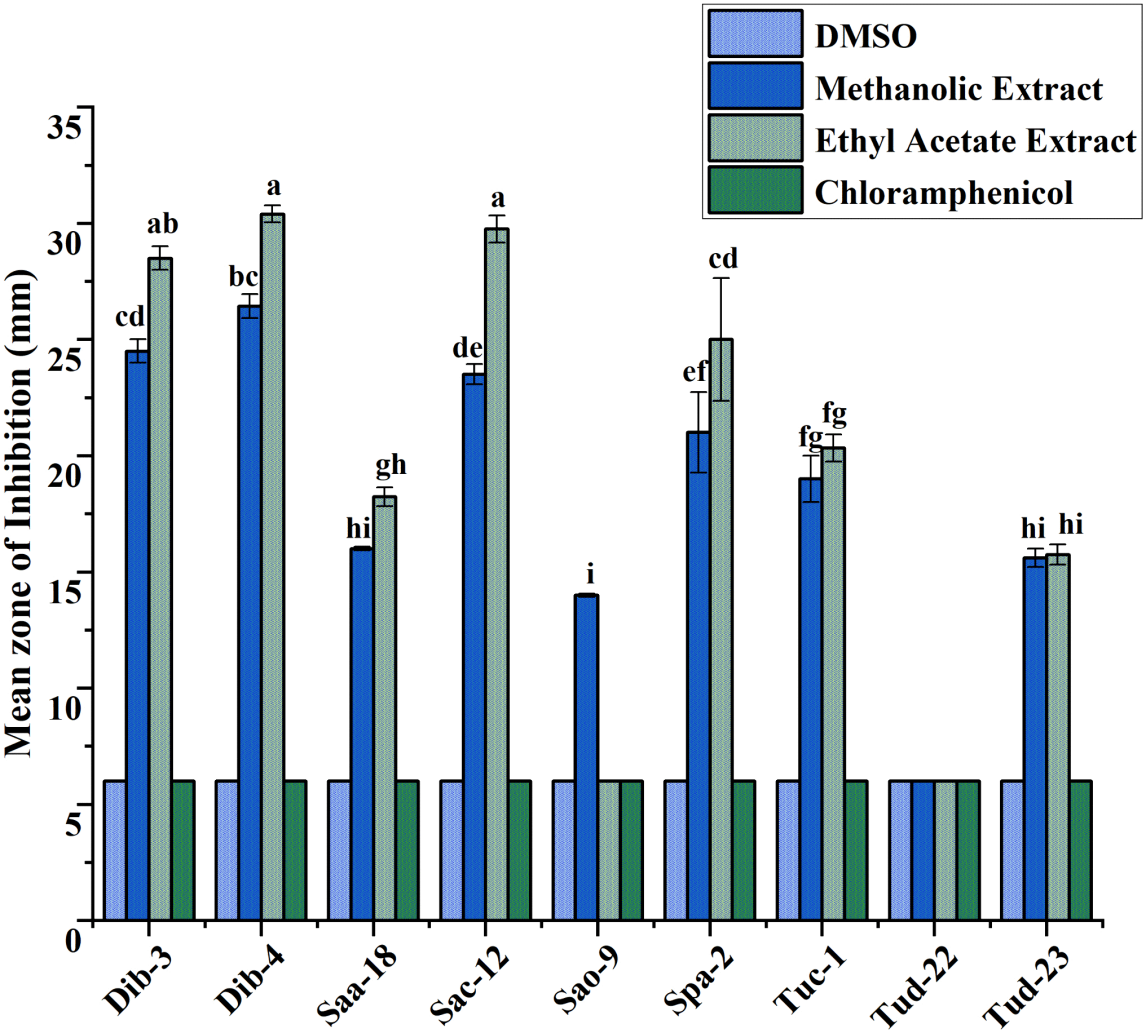
Antibacterial evaluation of Ethyl acetate and Methanol extracts of fungal epiphytes against drug resistant *P. aeruginosa* by the disc diffusion method. The values are the mean diameter (mm) of triplicate readings (mean ± SD; n = 3). Bars with different letters are significantly different according to Tukey’s post hoc test at p = 0.05. Therefore, any observed difference between them is considered to be statistically significant.

Following initial antibacterial activity screening, the metabolites of epiphytic fungal extracts of selected fungal crude extracts showing promising bioactive performance against test ESKAPE microorganisms were further evaluated using agar disc diffusion method. For reference, DMSO (negative control) and chloramphenicol (positive control) were included in the diffusion assays. As indicated in all the result graphs (Figs 4-9), both controls displayed inhibition zone diameters equivalent to the diameter of the paper disk (6 mm), which was plotted only for visualization and comparative purposes. Importantly, neither DMSO nor chloramphenicol produced any observable growth inhibition under the tested conditions, confirming the absence of inherent antibacterial activity in the solvent and the ineffectiveness of the commercially available chloramphenicol against these test pathogens [52].

### Antibacterial activity of crude fungal extracts against *E. cloacae*

The activity of crude extracts prepared using methanol and ethyl acetate was evaluated against drug resistant *E. cloacae*, and the results were presented as mean inhibition zones in Fig 4.

As illustrated in Fig 4, ethyl acetate extracts consistently demonstrated the highest antibacterial activity across all fungal isolates, with mean inhibition zones ranging from 15.33 to 19.33 mm. Notably, isolates Sac-12, Dib-4, Dib-3, and Tuc-1 exhibited the strongest activity, suggesting the presence of potent extracellular bioactive metabolites with broad-spectrum antibacterial potential. In comparison, methanolic extracts have showed moderate inhibitory effects, with inhibition zones ranging between 14.67 and 17.33 mm for most isolates, indicating that intracellular metabolites also contribute to antibacterial activity, albeit to a lesser extent. The results showed that ethyl acetate extracts are more effective than methanolic extracts in the case of suppressing *E. cloacae* growth, highlighting the efficiency of ethyl acetate as a solvent for extracting active secondary metabolites from these fungi. The marked difference between the two extraction solvents further underscores the importance of solvent polarity in recovering bioactive compounds.

### Antibacterial activity of crude fungal extracts against *A. baumannii*

The antibacterial activity of methanolic and ethyl acetate crude extracts from the evaluated fungal isolates against *A. baumannii* is summarized in Fig 5. Ethyl acetate extracts produced the largest inhibition zones overall (16.17–20.83 mm), whereas methanolic extracts yielded moderate activity ranging from 14.83–18.77 mm. In particular, isolates Dib-4, Tud-23, Dib-3, and Tuc-1 (17.33–20.83 mm), respectively have displayed the strongest activity (ethyl acetate greater than methanol), followed by Sac-12 and Tud-22 (16.17–16.80), which all produced clear, reproducible zones of inhibition. In contrast, Saa-18, Sao-9, and Spa-2 showed no antibacterial activity: mean inhibition diameters for these isolates were equal to the disk diameter (6 mm) for both solvents, indicating absence of diffusible inhibitory compounds under the extraction/assay conditions. All values are reported as mean ± SD of three independent replicates (n = 3). These results indicate that ethyl acetate is generally more effective than methanol at recovering antibacterial metabolites active against *A. baumannii*, while several isolates either lack such metabolites or produce non-diffusible/low-abundance compounds not detected by this assay.

### Antibacterial activity of crude fungal extracts against *E. faecium*

The inhibitory potential of epiphytic fungal extracts against *E. faecium* is presented in Fig 6. Depending on the fungal isolate, methanolic and ethyl acetate extracts exhibited strong antibacterial activity, with inhibition zones ranging from 15.17–23.00 mm and 16.00–25.17 mm, respectively. Among the isolates, Dib-4 displayed the most pronounced activity, particularly in the ethyl acetate extracts, which reached up to 25.17 mm. Moderate inhibition was recorded for Tud-22, Saa-18, Sac-12, Sao-9, Spa-2, and Tuc-1, while Tud-23 of both the extracts showed no detectable effect against *E. faecium*.

### Antibacterial activity of crude fungal extracts against *S. aureus*

The inhibitory effects of fungal extracts against drug-resistant *S. aureus* were shown in Fig 7. Methanolic and ethyl acetate extracts demonstrated strong antibacterial potential, with inhibition zones ranging from 15.17 to 28.67 mm. The highest activity was recorded for Dib-4 (28.67 mm) and Dib-3 (26.00 mm), particularly in methanolic extracts, followed by the better performance of ethyl acetate extracts of Tuc-1, Saa-18, Spa-2, Sac-12, and Tud-22, which showed consistent inhibition. Moderate activity was also observed for Sao-9 and Tud-23 extracts. Except Dib-4 and Dib-3, all other ethyl acetate extracts exhibited slightly stronger inhibition than methanolic extracts, highlighting their effectiveness in extracting potent metabolites against *S. aureus*.

### Antibacterial activity of crude fungal extracts against *K. pneumoniae*

The antibacterial assay against multidrug-resistant *K. pneumoniae* revealed that both methanolic and ethyl acetate fungal extracts exhibited remarkable inhibitory activity compared to the controls (Fig 8). Ethyl acetate extracts consistently produced the largest inhibition zones (14.17–18.75 mm), surpassing methanolic extracts, which also showed substantial activity (14.00–16.87 mm). In contrast, Saa-18 (ethyl acetate) and Tuc-1 (both ethyl acetate and methanolic) extracts exhibited no inhibitory effect; their disk diameters are displayed in the graph solely for visual reference. The results clearly demonstrate that bioactive metabolites present in the fungal extracts possess strong potential against *K. pneumoniae*, highlighting their promise as alternative antimicrobial sources against drug-resistant pathogens.

### Antibacterial activity of crude fungal extracts against *P. aeruginosa*

According to the result shown (Fig 9), both methanolic and ethyl acetate extracts produced significantly larger inhibition zones compared to the controls (DMSO and chloramphenicol). Among the extracts, the ethyl acetate solvent generally exhibited superior efficacy, with isolates Dib-4, Sac-12, and Dib-3 producing mean inhibition zones ranging 28.5–30.4 mm. Methanolic extracts also displayed strong activity, though generally lower than their ethyl acetate counterparts, with maximum inhibition observed also from isolates Dib-4, Dib-3, and Sac-12 (23.5–26.43 mm). In contrast, some extracts such as Tud-22 (both ethyl acetate and methanolic extracts) and Sao-9 (ethyl acetate) showed no inhibition, highlighting isolate-specific differences in bioactive metabolite production effective against multidrug-resistant *P. aeruginosa*.

The MIC and MBC data for the fungal crude extracts tested against ESKAPE pathogens were summarized (Table 6). MIC values spanned 0.078–2.5 mg/mL and MBC values 0.3125–10 mg/mL, depending on the property of the isolate and target organism. Several extracts showed potent, broad activity: Dib-4, Dib-3, and Sac-12 were consistently the most active across multiple pathogens. Noticeably, MICs as low as 0.078 mg/mL were recorded (Dib-4, Sac-12) against *P. aeruginosa*, while Dib-4 exhibited low MICs (0.3125 mg/mL) against *E. faecium* and *S. aureus*. Conversely, Saa-18, Sao-9, and Spa-2 were inactive against *A. baumannii*, Saa-18 and Tuc-1 showed no detectable activity against *K. pneumoniae*, and Tud-22 and Sao-9 failed to inhibit *P. aeruginosa* under the test conditions.

**Table 6:** Determination of MIC and MBC (mg/mL) of the ethyl acetate extracts of fungal epiphytes against the tested bacteria.

Types of Extracts	Test ESKAPE Microorganisms											
	*E. cloacae*		*A. baumannii*		*E. faecium*		*S. aureus*		*K. pneumoniae*		*P. aeruginosa*	
	MIC (mg/mL)	MBC (mg/mL)	MIC (mg/mL)	MBC (mg/mL)	MIC (mg/mL)	MBC (mg/mL)	MIC (mg/mL)	MBC (mg/mL)	MIC (mg/mL)	MBC (mg/mL)	MIC (mg/mL)	MBC (mg/mL)
Dib-3	1.25	2.5	1.25	2.5	1.25	2.5	0.625	1.25	1.25	2.5	0.156	0.625
Dib-4	1.25	1.25	0.625	1.25	0.3125	0.625	0.3125	0.625	1.25	1.25	0.078	0.3125
Saa-18	2.5	2.5	–	–	1.25	1.25	0.625	1.25	–	–	1.25	1.25
Sac-12	2.5	2.5	2.5	2.5	1.25	2.5	1.25	2.5	1.25	2.5	0.078	0.3125
Sao-9	2.5	10	–	–	2.5	5.0	1.25	2.5	2.5	5.0	–	–
Spa-2	1.25	2.5	–	–	1.25	2.5	0.625	1.25	2.5	5.0	0.3125	1.25
Tuc-1	2.5	2.5	2.5	2.5	1.25	1.25	0.625	1.25	–	–	0.625	1.25
Tud-22	2.5	5.0	2.5	5.0	0.625	1.25	1.25	2.5	2.5	2.5	–	–
Tud-23	2.5	5.0	2.5	5.0	–	–	2.5	5.0	2.5	5.0	2.5	2.5

(-) indicates no activity detected; MIC: Minimum inhibitory concentration; MBC: Minimum bactericidal concentration. Experiments were tested in triplicates. MIC and MBC values represent concentrations derived from a two-fold serial dilution series and are reported exactly as tested.

The minimum inhibitory and bactericidal activities of fungal extracts against ESKAPE test pathogens were summarized (Table 7). Overall, MIC values ranged from 0.156 to 2.5 mg/mL, while MBC values extended from 0.3125 to 10 mg/mL, indicating variability in potency across isolates and target pathogens. Among the extracts, Dib-4 consistently demonstrated the strongest and broadest activity, with MICs as low as 0.156 mg/mL against *S. aureus* and *P. aeruginosa* and MBCs of 0.3125 mg/mL, highlighting potent bactericidal potential. Similarly, Dib-3 displayed strong effects against *S. aureus* (MIC 0.156 mg/mL, MBC 0.3125 mg/mL) and *E. faecium* (MIC 1.25 mg/mL, MBC 2.5 mg/mL). Moderate activity was observed for Sac-12 and Spa-2, particularly against *P. aeruginosa* (MICs 0.3125–0.625 mg/mL). However, Saa-18, Sao-9, Tuc-1, Tud-22, and Tud-23 exhibited relatively weaker or inconsistent activity, with higher MIC/MBC values (up to 2.5/10 mg/mL) or no detectable inhibition against certain test microorganisms.

**Table 7:** Determination of MIC and MBC (mg/mL) of the methanolic extracts of fungal epiphytes against the tested bacteria.

Types of Extracts	Test ESKAPE Microorganisms											
	*E. cloacae*		*A. baumannii*		*E. faecium*		*S. aureus*		*K. pneumoniae*		*P. aeruginosa*	
	MIC (mg/mL)	MBC (mg/mL)	MIC (mg/mL)	MBC (mg/mL)	MIC (mg/mL)	MBC (mg/mL)	MIC (mg/mL)	MBC (mg/mL)	MIC (mg/mL)	MBC (mg/mL)	MIC (mg/mL)	MBC (mg/mL)
Dib-3	2.5	2.5	2.5	2.5	1.25	2.5	0.156	0.3125	2.5	5.0	0.3125	0.625
Dib-4	1.25	2.5	1.25	2.5	0.3125	0.3125	0.156	0.3125	2.5	2.5	0.156	0.3125
Saa-18	2.5	5.0	–	–	2.5	2.5	1.25	2.5	2.5	5.0	2.5	2.5
Sac-12	2.5	2.5	2.5	2.5	1.25	2.5	2.5	2.5	2.5	2.5	0.3125	0.625
Sao-9	2.5	10.0	–	–	2.5	5.0	2.5	5.0	2.5	5.0	2.5	10.0
Spa-2	2.5	5.0	–	–	1.25	5.0	1.25	5.0	2.5	5.0	0.625	2.5
Tuc-1	2.5	5.0	2.5	5.0	2.5	2.5	1.25	2.5	–	–	1.25	2.5
Tud-22	2.5	5.0	2.5	5.0	1.25	2.5	1.25	2.5	2.5	5.0	–	–
Tud-23	2.5	5.0	2.5	10.0	–	–	2.5	5.0	2.5	5.0	2.5	5.0

(-) indicates no activity detected; MIC: Minimum inhibitory concentration; MBC: Minimum bactericidal concentration. Experiments were performed in triplicates. MIC and MBC values represent concentrations derived from a two-fold serial dilution series and are reported exactly as tested.

The MIC and MBC performances of epiphytic fungal crude extracts prepared with methanol and ethyl acetate (combined) against the test pathogens were demonstrated (Table 8). MIC values ranged from 0.039 to 2.5 mg/mL, while MBC values varied between 0.156 and 10 mg/mL. This clearly reflecting differences in potency across isolates and pathogens. Dib-4 consistently exhibited the strongest and broadest activity, with remarkably low MIC/MBC values of 0.156/0.156 mg/mL against *E. faecium* and *S. aureus*, and as low as 0.039/0.156 mg/mL against *P. aeruginosa*. Likewise, Dib-3 and Sac-12 has also showed strong effects, with MICs of 0.156–0.625 mg/mL against *S. aureus* and *E. faecium*, and 0.078 mg/mL *P. aeruginosa*. Moderate activity was recorded for Spa-2, Tuc-1, and Tud-22, with MICs generally between 0.3125 to 1.25 mg/mL and MBCs of 1.25 to 2.5 mg/mL, while Saa-18, Sao-9, and Tud-23 displayed relatively weaker effects, with higher MIC/MBC values (up to 2.5/10 mg/mL) and inactivity against some of the test pathogens.

**Table 8:** Determination of MIC and MBC (mg/mL) of the combined extracts of fungal epiphytes against the tested bacteria.

Types of Extracts	Test ESKAPE Microorganisms											
	*E. cloacae*		*A. baumannii*		*E. faecium*		*S. aureus*		*K. pneumoniae*		*P. aeruginosa*	
	MIC (mg/mL)	MBC (mg/mL)	MIC (mg/mL)	MBC (mg/mL)	MIC (mg/mL)	MBC (mg/mL)	MIC (mg/mL)	MBC (mg/mL)	MIC (mg/mL)	MBC (mg/mL)	MIC (mg/mL)	MBC (mg/mL)
Dib-3	1.25	1.25	1.25	1.25	0.625	1.25	0.156	0.3125	1.25	2.5	0.078	0.3125
Dib-4	0.625	0.625	0.625	0.625	0.156	0.156	0.156	0.156	1.25	1.25	0.039	0.156
Saa-18	2.5	2.5	–	–	1.25	1.25	1.25	1.25	2.5	5.0	1.25	1.25
Sac-12	1.25	1.25	1.25	1.25	0.625	1.25	1.25	1.25	1.25	2.5	0.078	0.3125
Sao-9	1.25	5.0	–	–	1.25	5.0	1.25	5.0	2.5	5.0	2.5	10.0
Spa-2	2.5	2.5	–	–	1.25	2.5	0.625	1.25	1.25	2.5	0.156	0.625
Tuc-1	1.25	2.5	1.25	2.5	1.25	2.5	0.3125	1.25	–	–	0.625	1.25
Tud-22	2.5	5.0	1.25	2.5	0.625	1.25	0.625	1.25	2.5	2.5	–	–
Tud-23	1.25	2.5	1.25	2.5	–	–	1.25	2.5	2.5	5.0	2.5	5.0

(-) indicates no activity detected; MIC: Minimum inhibitory concentration; MBC: Minimum bactericidal concentration. Experiments were performed in triplicates. MIC and MBC values represent concentrations derived from a two-fold serial dilution series and are reported exactly as tested.

The mode of action of ethyl acetate, methanolic, and combined fungal extracts showed bactericidal (BC) activity (MBC/MIC ≤ 4) against the tested microorganisms (Table 9). The ethyl acetate extracts demonstrated consistent bactericidal effects across nearly all host isolates; with only occasional absence of activity. Similarly, methanolic extracts showed broad-spectrum bactericidal potential, although some fungal isolates exhibited no effect against specific pathogens. The synergistic combinations generally maintained or improved bactericidal (BC) efficacy, with several isolates (Dib-3, Dib-4, and Sac-12) showing strong and consistent activity across all the pathogens tested in the present study. However, a few combinations still displayed no detectable activity against certain microorganisms.

**Table 9:** Summarizes the mode of action of ethyl acetate, methanolic, and combined fungal extracts against ESKAPE bacteria.

Types of Extracts	Mode of action (Ethyl acetate extract)											
	*E. cloacae*		*A. baumannii*		*E. faecium*		*S. aureus*		*K. pneumoniae*		*P. aeruginosa*	
	MBC/MIC	Mode of Action	MBC/MIC	Mode of action	MBC/MIC	Mode of action	MBC/MIC	Mode of action	MBC/MIC	Mode of action	MBC/MIC	Mode of action
Dib-3	2	BC	2	BC	2	BC	2	BC	2	BC	4	BC
Dib-4	1	BC	2	BC	2	BC	2	BC	1	BC	4	BC
Saa-18	1	BC	–	–	1	BC	2	BC	–	–	1	BC
Sac-12	1	BC	1	BC	2	BC	2	BC	2	BC	4	BC
Sao-9	4	BC	–	–	2	BC	2	BC	2	BC	–	–
Spa-2	2	BC	–	–	2	BC	2	BC	2	BC	4	BC
Tuc-1	1	BC	1	BC	1	BC	2	BC	–	–	2	BC
Tud-22	2	BC	2	BC	2	BC	2	BC	1	BC	–	–
Tud-23	2	BC	2	BC	–	–	2	BC	2	BC	1	BC
**Mode of action (Methanolic extract)**												
Dib-3	1	BC	1	BC	2	BC	2	BC	2	BC	2	BC
Dib-4	2	BC	2	BC	1	BC	2	BC	1	BC	2	BC
Saa-18	2	BC	–	–	1	BC	2	BC	2	BC	1	BC
Sac-12	1	BC	1	BC	2	BC	2	BC	1	BC	2	BC
Sao-9	4	BC	–	–	2	BC	2	BC	2	BC	4	BC
Spa-2	2	BC	–	–	4	BC	4	BC	2	BC	4	BC
Tuc-1	2	BC	2	BC	1	BC	2	BC	–	–	2	BC
Tud-22	2	BC	2	BC	2	BC	2	BC	2	BC	–	–
Tud-23	2	BC	4	BC	–	–	2	BC	2	BC	2	BC
**Mode of action (Combined)**												
Dib-3	1	BC	1	BC	2	BC	2	BC	2	BC	4	BC
Dib-4	1	BC	1	BC	1	BC	1	BC	1	BC	4	BC
Saa-18	1	BC	–	–	1	BC	1	BC	2	BC	1	BC
Sac-12	1	BC	1	BC	2	BC	1	BC	2	BC	4	BC
Sao-9	4	BC	–	–	4	BC	4	BC	2	BC	4	BC
Spa-2	1	BC	–	–	2	BC	2	BC	2	BC	4	BC
Tuc-1	2	BC	2	BC	2	BC	4	BC	–	–	2	BC
Tud-22	2	BC	2	BC	2	BC	2	BC	1	BC	–	–
Tud-23	2	BC	2	BC	–	–	2	BC	2	BC	2	BC

(-) indicates no activity detected; MIC: Minimum inhibitory concentration; MBC: Minimum bactericidal concentration. Bactericidal (BC) effect: MBC/MIC ≤ 4, indicating a predominantly bactericidal profile for the active extracts.

### Mechanism of action

#### Morphological changes of the bacterial cells after extract treatment.

**Fig 10:**
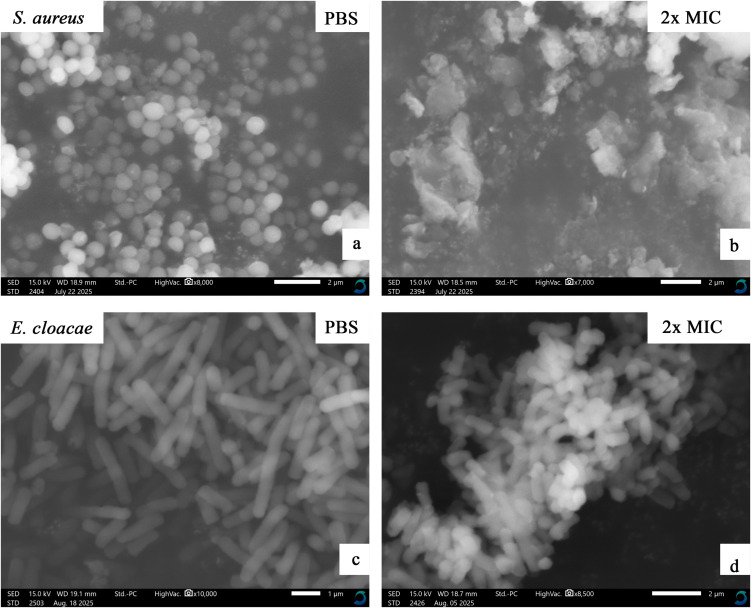
SEM micrographs showing the effects of Dib-3 extract (combined) at the concentration of 2x MIC mg/mL on *S. aureus* and *E. cloacae* cells. (a) and (c) are 1x PBS-treated cells (controls). (b) and (d) are extract-treated cells of the test bacteria.

**Fig 11:**
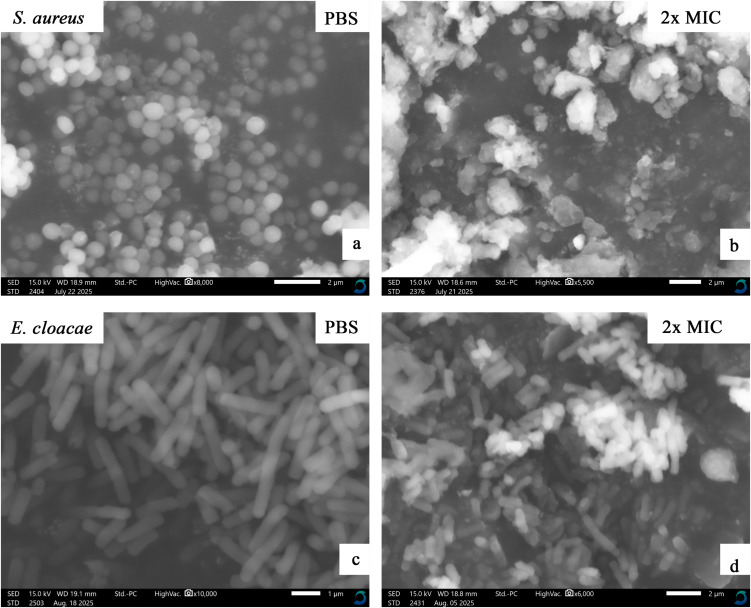
SEM micrographs showing the effects of Dib-4 extract (combined) at the concentration of 2x MIC mg/mL on *S. aureus* and *E. cloacae* cells. (a) and (c) are 1x PBS-treated cells (controls). (b) and (d) are extract-treated cells of the test bacteria.

**Fig 12:**
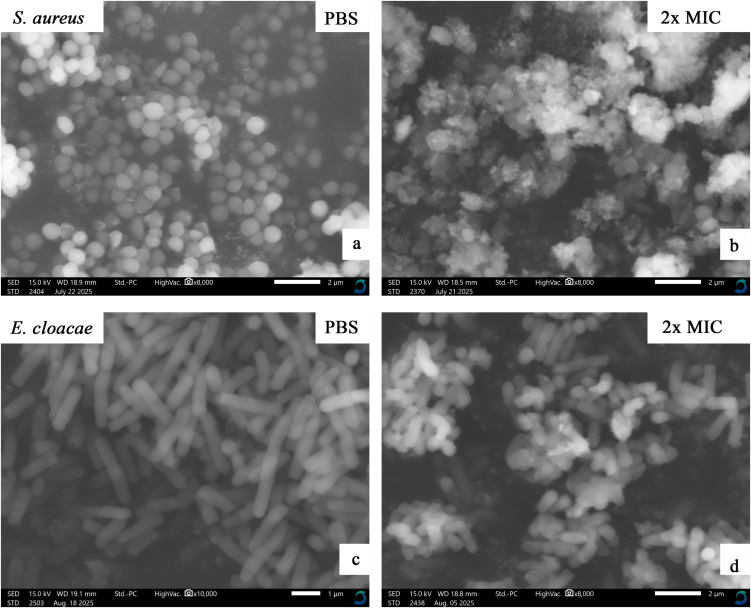
SEM micrographs showing the effects of Sac-12 extract (combined) at the concentration of 2x MIC mg/mL on *S. aureus* and *E. cloacae* cells. (a) and (c) are 1x PBS-treated cells (controls). (b) and (d) are extract-treated cells of the test bacteria.

The effects of Dib-3 (combined), Dib-4 (combined), and Sac-12 (combined) extracts on bacterial cells were evaluated using SEM, with the corresponding results presented in Figs 10–12, respectively. Figs 10a, 11a, and 12a were the controls (PBS-treated) cells of *S. aureus* which displayed the intact cocci shaped with smooth cell surface and still maintained cellular rigidity. However, after 12 hours of exposure to the extracts, the bacterial cells start to lose their intact cocci shape, lysed and shrunk abruptly (Figs 10b, 11b, and 12b).

Similarly, Figs 10c, 11c, and 12c, respectively, shows the micrographs of a control and treated of Gram-negative bacterial cells, *E. cloacae* before and after treatment with extracts. The controls (PBS-treated treated) cells were existed in normal cell condition with rugose surface, rod shape rigid and smooth cell surface. However, after the cells were treated with the extracts for overnight, significant morphological changes of the cells could be observed including the invagination and formation of cavities on the cell surfaces. The SEM analyses showed that the extract-treated cells had undergone severe structural damages which finally caused the cell membrane to breakdown (Figs 10d, 11d, and 12d).

### Gas chromatography-coupled mass spectrometry (GC-MS) analysis

**Fig 13:**
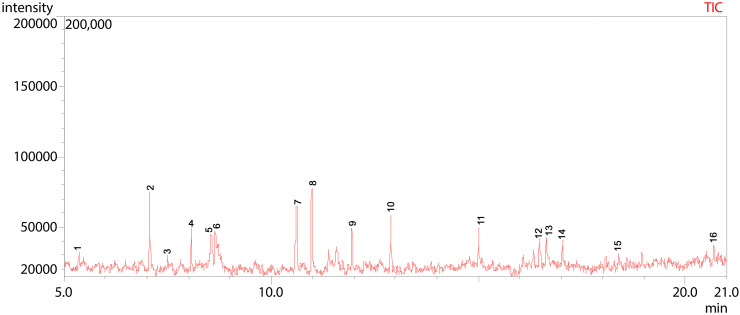
GC-MS chromatogram illustration of detected compounds in ethyl acetate extract of Dib-3. Numbers above the Peaks represent the individual chemical components.

**Fig 14:**
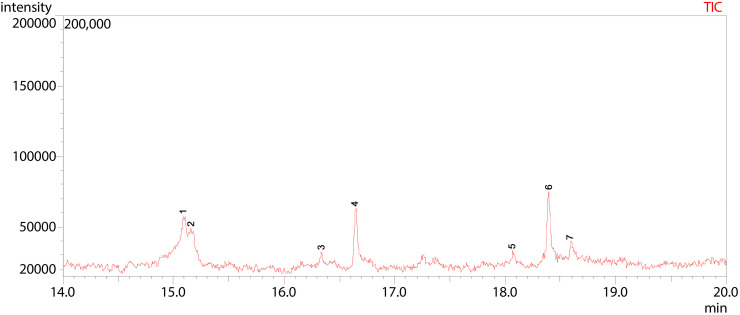
GC-MS chromatogram illustration of detected compounds in methanolic of Dib-3. Numbers above the Peaks represent the individual chemical components.

**Fig 15:**
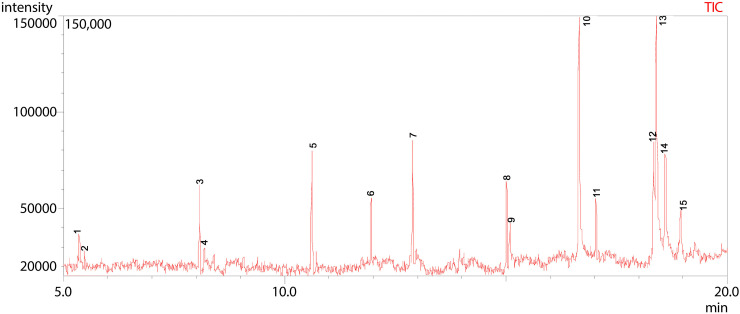
GC-MS chromatogram illustration of detected compounds in ethyl acetate extract of Dib-4. Numbers above the Peaks represent the individual chemical components.

**Fig 16:**
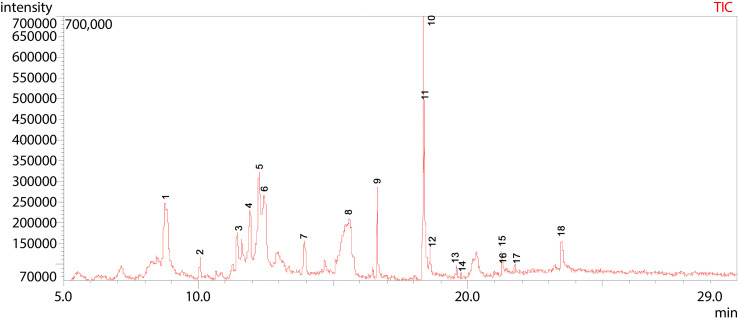
GC-MS chromatogram illustration of detected compounds in methanolic extract of Dib-4. Numbers above the Peaks represent the individual chemical components.

**Fig 17:**
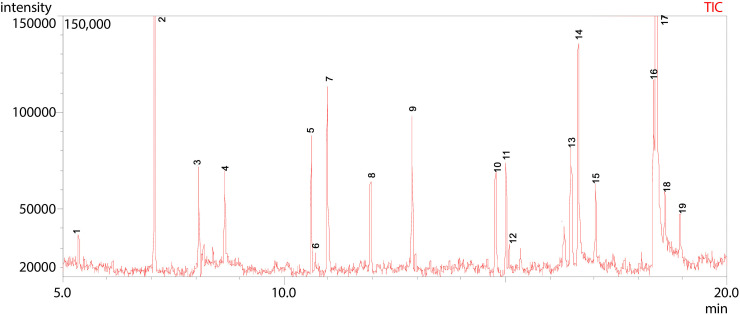
GC-MS chromatogram illustration of detected compounds in ethyl acetate extract of Sac-12. Numbers above the Peaks represent the individual chemical components.

**Fig 18:**
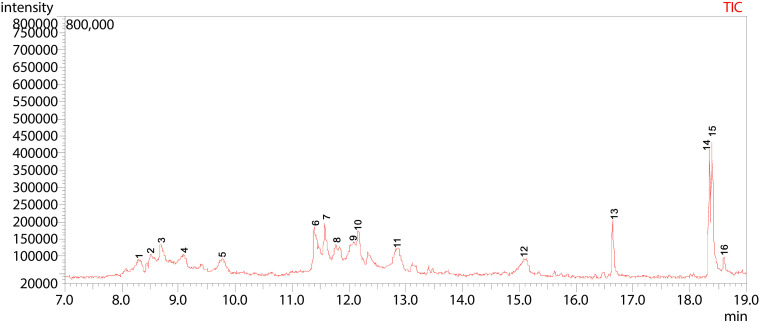
GC-MS chromatogram illustration of detected compounds in methanolic extract of Sac-12. Numbers above the Peaks represent the individual chemical components.

Gas chromatography-mass spectrometry (GC-MS) analysis of bioactive compounds was performed on three selected fungal extracts, namely Dib-3, Dib-4, and Sac-12. It focused on these representative extracts to ensure a targeted and resource-efficient analytical approach. The extracts were prioritized based on their broad-spectrum antibacterial efficacy against all tested drug-resistant bacterial strains and their pronounced membrane-disruptive effects as revealed by scanning electron microscopy (SEM) analysis. This enabled the characterization of major volatile and semi-volatile metabolites potentially responsible for the observed antibacterial and mechanistic effects, thereby providing critical insights into the mode of action of these fungal-derived compounds. Chemical components identified in the brown seaweed-associated epiphytic fungi, Dib-3, Dib-4, and Sac-12 by GC-MS analyses were shown in Figs 13-18 and Tables 10-15.

**Table 10:** GC-MS-based secondary metabolite profiling of the Dib-3 ethyl acetate extract.

Sample	Peak Nr.	R_T_ (min)	Name of identified compound	Peak Area %	MW (g/mol)	Molecular Formula
Dib-3	1	5.355	1-Decene	2.02	140	C_10_H_20_
	2	7.069	Phenylethyl Alcohol	10.47	122	C_8_H_10_O
	3	7.501	Cyclobutane-1,1-dicarboxamide, N,N’-di-benzoyloxy-	1.80	382	C_20_H_18_N_2_O_6_
	4	8.075	1-Dodecene	4.92	168	C_12_H_24_
	5	8.548	Cyclohexyl dimethyl isopropoxysilane	8.53	200	C_11_H_24_OSi
	6	8.649	Benzeneacetic acid	5.52	136	C_8_H_8_O_2_
	7	10.615	1-Tridecene	11.14	182	C_13_H_26_
	8	10.979	Benzeneethanol, 4-hydroxy-	13.61	138	C_8_H_10_O_2_
	9	11.952	2,4-Di-tert-butylphenol	5.28	206	C_14_H_22_O
	10	12.890	Tetradecyl trifluoroacetate	8.63	310	C_16_H_29_F_3_O_2_
	11	15.012	Z-5-Nonadecene	6.71	266	C_19_H_38_
	12	16.484	Pyrrolo[1,2-a]pyrazine-1,4-dione, hexahydro-3-(2-methylpropyl)-	6.65	210	C_11_H_18_N_2_O_2_
	13	16.644	l-(+)-Ascorbic acid 2,6-dihexadecanoate	5.73	652	C_38_H_68_O_8_
	14	17.036	n-Tetracosanol-1	4.98	354	C_24_H_50_O
	15	18.387	Oxacyclopentadecan-2-one	1.98	226	C_14_H_26_O_2_
	16	20.686	Diisooctyl adipate	2.03	370	C_22_H_42_O_4_

**Table 15:** GC-MS-based secondary metabolite profiling of the Sac-12 methanolic extract.

Sample	Peak Nr.	R_T_ (min)	Compound identified	Peak Area %	MW (g/mol)	Molecular Formula
Sac-12	1	8.306	Isosorbide	4.86	146	C_6_H_10_O_4_
	2	8.506	Dianhydromannitol	4.40	146	C_6_H_10_O_4_
	3	8.688	Acetic acid, TBDMS derivative	4.23	174	C_8_H_18_O_2_Si
	4	9.098	Erythritol	2.24	122	C_4_H_10_O_4_
	5	9.766	2,5-Methylene-d,l-rhamnitol	7.10	178	C_7_H_14_O_5_
	6	11.391	Dimethyl(bis[(2Z)-pent-2-en-1-yloxy]) silane	7.99	228	C_12_H_24_O_2_Si
	7	11.575	Silane, [(1,1-dimethyl-2-propenyl)oxy]dimethyl-	6.68	144	C_7_H_16_OSi
	8	11.771	1,4-Anhydro-d-galactitol	5.61	164	C_6_H_12_O_5_
	9	12.035	D-glycero-D-manno-Heptitol	2.94	212	C_7_H_16_O_7_
	10	12.170	d-Mannitol, 1,4-anhydro-	4.28	164	C_6_H_12_O_5_
	11	12.867	1,5-Anhydro-d-altritol	6.74	164	C_6_H_12_O_5_
	12	15.115	D-Mannitol	4.17	182	C_6_H_14_O_6_
	13	16.641	l-(+)-Ascorbic acid 2,6-dihexadecanoate	6.27	652	C_38_H_68_O_8_
	14	18.341	Linoelaidic acid	12.34	280	C_18_H_32_O_2_
	15	18.392	cis-10-Heptadecenoic acid	18.17	268	C_17_H_32_O_2_
	16	18.598	Octadecanoic acid	1.99	284	C_18_H_36_O_2_

As indicated in Fig 13 and Table 10, the total ion chromatogram (TIC) from GC-MS analysis of Dib-3 displayed 16 distinct peaks within a retention time range of 5.36 to 20.69 minutes, indicating the presence of 16 different volatile and semi-volatile compounds. The major constituents include Phenylethyl Alcohol (peak 2), Cyclohexyl dimethyl isopropoxysilane (peak 5), 1-Tridecene (peak 7), Benzeneethanol, 4-hydroxy- (peak 8), and Tetradecyl trifluoroacetate (peak 10), suggesting that they are the most abundant compounds in the ethyl acetate extract of Dib-3. Several minor peaks of lower intensity appeared between 5 and 10 minutes and between 12 and 21 minutes, corresponding to less abundant components.

The GC-MS total ion chromatogram (TIC) of the methanolic extract of Dib-3 displayed seven [7] well-resolved peaks within a retention time range of 15.09 to 19.00 minutes, supporting the presence of seven different volatile and semi-volatile compounds. The dominant constituents were 6-azathymine, bis(methyl) ether (peak 1), D-mannitol (peak 2), l-(+)-ascorbic acid 2,6-dihexadecanoate (peak 4), and cis-vaccenic acid (peak 6), indicating their high relative most abundance in the extract. In contrast, hexadecanoic acid, methyl ester (peak 3), 9-octadecenoic acid (Z)-, methyl ester (peak 5), and octadecanoic acid (peak 7) were appeared with minor peaks of lower intensity corresponding to less abundant components (Fig 14 and Table 11).

**Table 11:** GC-MS-based secondary metabolite profiling of the Dib-3 methanolic extract.

Sample	Peak Nr.	R_T_ (min)	Name of identified compound	Peak Area %	MW (g/mol)	Molecular Formula
Dib-3	1	15.091	6-Azathymine, Bis(methyl) ether	34.53	155	C_6_H_9_N_3_O_2_
	2	15.161	D-Mannitol	16.48	182	C_6_H_14_O_6_
	3	16.341	Hexadecanoic acid, methyl ester	2.05	270	C_17_H_34_O_2_
	4	16.648	l-(+)-Ascorbic acid 2,6-dihexadecanoate	27.53	652	C_38_H_68_O_8_
	5	18.067	9-Octadecenoic acid (Z)-, methyl ester	2.35	296	C_19_H_36_O_2_
	6	18.393	cis-Vaccenic acid	12.53	282	C_18_H_34_O_2_
	7	18.599	Octadecanoic acid	4.52	284	C_18_H_36_O_2_

According to the total ion chromatogram (TIC) obtained from GC-MS analysis, the ethyl acetate extract of Dib-4 has showed a complex profile with 15 peaks distributed over a retention time range of 5.36–18.95 min (Fig 15 and Table 12). A total of 15 compounds were identified, of which Pentadecanoic acid (18.14%) at peak 10 and cis-10-Heptadecenoic acid (21.07%) at peak 13 dominated the extract. Similarly, Linoelaidic acid (8.74%) at peak 12, Octadecanoic acid (7.94%) at peak 14, 1-Tetradecene (7.22%) at peak 5, and 9-Eicosene, (E)- (7.12%) at peak 7 were also detected as prominent constituents, apart from other minor components such as 1-Heneicosanol (5.63%), 1-Dodecene (5.05%), and 2,4-Di-tert-butylphenol (4.05%) in the extract.

**Table 12:** GC-MS-based secondary metabolite profiling of the Dib-4 ethyl acetate extract.

Sample	Peak Nr.	R_T_ (min)	Name of identified compound	Peak Area %	MW (g/mol)	Molecular Formula
Dib-4	1	5.355	Cyclopropane, nonyl-	1.16	168	C_12_H_24_
	2	5.472	Nonane	1.22	128	C_9_H_20_
	3	8.075	1-Dodecene	5.05	168	C_12_H_24_
	4	8.184	Dodecane	1.57	170	C_12_H_26_
	5	10.613	1-Tetradecene	7.22	196	C_14_H_28_
	6	11.953	2,4-Di-tert-butylphenol	4.05	206	C_14_H_22_O
	7	12.891	9-Eicosene, (E)-	7.12	280	C_20_H_40_
	8	15.016	1-Heneicosanol	5.63	312	C_21_H_44_O
	9	15.091	6-Azathymine, Bis(methyl) ether	3.66	155	C_6_H_9_N_3_O_2_
	10	16.652	Pentadecanoic acid	18.14	242	C_15_H_30_O_2_
	11	17.038	Trifluoroacetic acid, pentadecyl ester	3.49	324	C_17_H_31_F_3_O_2_
	12	18.346	Linoelaidic acid	8.74	280	C_18_H_32_O_2_
	13	18.397	cis-10-Heptadecenoic acid	21.07	268	C_17_H_32_O_2_
	14	18.604	Octadecanoic acid	7.94	284	C_18_H_36_O_2_
	15	18.949	n-Nonadecanol-1	3.94	284	C_19_H_40_O

The chemical profile of Dib-4 (methanolic extract) was analyzed using GC-MS, and the total ion chromatogram (TIC) revealed a chemically diverse composition with resolved peaks eluting over a retention time range of 8.76–23.49 min (Fig 16 and Table 13). A total of 18 secondary metabolites were tentatively identified. The major constituents of the extract were d-Mannitol, 1,4-anhydro- (13.02%) at peak 5, DL-Arabinitol (16.60%) at peak 6, Linoelaidic acid (13.34%) at peak 10, and 22-Tricosenoic acid (13.36%) at peak 11. In addition, moderate levels of 1-Methyl-pyrrolidine-2-carboxylic acid (10.58%) at peak 1 and 3,4-Altrosan (6.97%) at peak 4 were detected, along with other several minor components such as Dimethyl(bis[(2Z)-pent-2-en-1-yloxy]) silane (peak 3), 3-Deoxy-d-mannitol (peak 7), and l-(+)-Ascorbic acid 2,6-dihexadecanoate (peak 9).

**Table 13:** GC-MS-based secondary metabolite profiling of the Dib-4 methanolic extract.

Sample	Peak Nr.	R_T_ (min)	Compound identified	Peak Area %	MW (g/mol)	Molecular Formula
Dib-4	1	8.763	1-Methyl-pyrrolidine-2-carboxylic acid	10.58	129	C_6_H_11_NO_2_
	2	10.067	2,5-Monomethylene-l-rhamnitol	1.51	178	C_7_H_14_O_5_
	3	11.430	Dimethyl(bis[(2Z)-pent-2-en-1-yloxy]) silane	4.42	228	C_12_H_24_O_2_Si
	4	11.928	3,4-Altrosan	6.97	162	C_6_H_10_O_5_
	5	12.253	d-Mannitol, 1,4-anhydro-	13.02	164	C_6_H_12_O_5_
	6	12.466	DL-Arabinitol	16.60	152	C_5_H_12_O_5_
	7	13.957	3-Deoxy-d-mannitol	4.59	166	C_6_H_14_O_5_
	8	15.646	D-Mannitol	2.42	182	C_6_H_14_O_6_
	9	16.640	l-(+)-Ascorbic acid 2,6-dihexadecanoate	4.58	652	C_38_H_68_O_8_
	10	18.339	Linoelaidic acid	13.34	280	C_18_H_32_O_2_
	11	18.388	22-Tricosenoic acid	13.36	352	C_23_H_44_O_2_
	12	18.592	Octadecanoic acid	2.52	284	C_18_H_36_O_2_
	13	19.583	Pyrrolo[1,2-a]pyrazine-3-propanamide, 2,3,6,7,8,8a-hexahydro-1,4-dioxo-	1.11	225	C_10_H_15_N_3_O_3_
	14	19.756	Fumaric acid, 2-dimethylaminoethyl octadecyl ester	0.22	439	C_26_H_49_NO_4_
	15	21.268	3-Cyclopentylpropionic acid, 2-dimethylaminoethyl ester	1.17	213	C_12_H_23_NO_2_
	16	21.303	S-[2-[N,N-Dimethylamino]ethyl]morpholine-N-carbonylthiocarbohydroximate	0.37	261	C_10_H_19_N_3_O_3_S
	17	21.750	Hexadecanoic acid, 2-hydroxy-1-(hydroxymethyl)ethyl ester	0.42	330	C_19_H_38_O_4_
	18	23.491	Butyl 9,12-octadecadienoate	2.77	336	C_22_H_40_O_2_

The total ion chromatography (TIC) of ethyl acetate extract of Sac-12 revealed a chemically diverse profile with 19 peaks eluted within a retention time range of 5.36–18.95 min. A total of 19 compounds that collectively accounted for 100% of the total peak area were identified (Fig 17 and Table 14). The extract was highly dominated by cis-10-Heptadecenoic acid (38.40%) at peak 17, followed by Phenylethyl Alcohol (9.12%) at peak 2, and l-(+)-Ascorbic acid 2,6-dihexadecanoate (6.30%) at peak 14. Other notable metabolites such as Benzeneethanol, 4-hydroxy- (6.03%) at peak 7, Linoelaidic acid (5.67%) at peak 16, and 1-Tridecene (3.44%) at peak 5 were detected as minor metabolites in this particular extract.

**Table 14:** GC-MS-based secondary metabolite profiling of the Sac-12 ethyl acetate extract.

Sample	Peak Nr.	R_T_ (min)	Compound identified	Peak Area %	MW (g/mol)	Molecular Formula
Sac-12	1	5.356	1-Decene	0.41	140	C_10_H_20_
	2	7.070	Phenylethyl Alcohol	9.12	122	C_8_H_10_O
	3	8.076	1-Dodecene	2.69	168	C_12_H_24_
	4	8.656	Benzeneacetic acid	2.48	136	C_8_H_8_O_2_
	5	10.613	1-Tridecene	3.44	182	C_13_H_26_
	6	10.705	Decane, 2,3,5-trimethyl-	0.87	184	C_13_H_28_
	7	10.978	Benzeneethanol,4-hydroxy-	6.03	138	C_8_H_10_O_2_
	8	11.951	2,4-Di-tert-butylphenol	2.47	206	C_14_H_22_O
	9	12.890	Tetradecyl trifluoroacetate	3.34	310	C_16_H_29_F_3_O_2_
	10	14.775	Tryptophol	3.34	161	C_10_H_11_NO
	11	15.013	Z-5-Nonadecene	2.80	266	C_19_H_38_
	12	16.323	L-Proline, N-valeryl-, heptadecyl ester	0.97	437	C_27_H_51_NO_3_
	13	16.478	Pyrrolo[1,2-a]pyrazine-1,4-dione, hexahydro-3-(2-methylpropyl)-	5.03	210	C_11_H_18_N_2_O_2_
	14	16.647	l-(+)-Ascorbic acid 2,6-dihexadecanoate	6.30	652	C_38_H_68_O_8_
	15	17.035	9-Tricosene, (Z)-	2.18	322	C_23_H_46_
	16	18.346	Linoelaidic acid	5.67	280	C_18_H_32_O_2_
	17	18.400	cis-10-Heptadecenoic acid	38.40	268	C_17_H_32_O_2_
	18	18.601	Octadecanoic acid	3.25	284	C_18_H_36_O_2_
	19	18.947	1-Heptacosanol	1.32	396	C_27_H_56_O

As illustrated in Fig 18 and Table 15 below, the gas chromatography-mass spectrometry (GC-MS) total ion chromatogram (TIC) of Sac-12 (methanolic extract) showed a complex metabolite profile with 16 resolved peaks eluted over a retention time range of 8.31–18.60 min, corresponding to 16 identified volatile and semi volatile compounds. The major composition was contributed by cis-10-Heptadecenoic acid (18.17%) at peak 15 and Linoelaidic acid (12.34%) at peak 14, followed by Dimethyl(bis[(2Z)-pent-2-en-1-yloxy]) silane (7.99%) at peak 6, 2,5-Methylene-d,l-rhamnitol (7.10%) at peak 5, 1,5-Anhydro-d-altritol (6.74%) at peak 11, and Silane, [(1,1-dimethyl-2-propenyl)oxy]dimethyl- (6.68%) at peak 7. Other compounds such as Octadecanoic acid (1.99%) at peak 16, Erythritol (2.24%) at peak 4, and D-glycero-D-manno-Heptitol (2.94%) at peak 9 were detected as minor constituents.

## Discussion

Marine-derived fungi remain one of the most underexplored microbial groups, despite their well-documented ability to synthesize structurally diverse and biologically active metabolites with pharmaceutical potential [53]. In particular, marine algal-associated fungal symbionts are increasingly recognized as powerhouses of novel bioactive compounds, making them promising candidates for eco-friendly drug discovery [54]. The current study represents the first report on the isolation and antibacterial evaluation of fungal epiphytes from brown algae along the Kenyan coast, addressing an important gap in fungal bioprospecting within the region. Morphological characterization coupled with ITS-based molecular analysis confirmed that the isolates predominantly belonged to six metabolite-rich genera: *Fusarium*, *Alternaria*, *Aspergillus*, *Curvularia*, *Penicillium*, and *Talaromyces*—all of which are prolific producers of antimicrobial, cytotoxic, and antifungal compounds [55,56]. Notably, several isolates clustered robustly with reference strains known for producing a wide range of metabolites, including polyketides, thereby strengthening the likelihood that the observed bioactivities in this study are attributable to chemically diverse secondary metabolites encoded within their biosynthetic gene clusters [57]. This was also very similar to what has been described in other culture-dependent studies on marine fungi associated with brown algae [58].

The antibacterial activity of fungal epiphytes was preliminary screened by opting agar plug diffusion assay as this method was only used to detect the presence of antibacterial compounds secreted by the fungal agar plugs. In a meantime, the disc diffusion assay method was selected to precisely quantify the antibacterial activity of fermentative broth and fungal mycelium as this method had been acknowledged as a wise choice for the secondary screening stage [59]. Naturally, fungal epiphytes tend to secrete their bioactive metabolites extracellularly into the fermentation medium as the function of these metabolites is to keep them save from predators or pathogens. All fungal extracts screened in this study demonstrated remarkable antibacterial activity, with both ethyl acetate and methanolic fractions inhibiting the growth of tested bacteria, indicating that the active metabolites were distributed across both intracellular and extracellular compartments. The ethyl acetate extracts generally yielded broader and stronger inhibition across most pathogens, while the methanolic extracts produced the largest inhibition zones for isolates Dib-3 and Dib-4 specifically against drug-resistant *S. aureus*. These findings align with previous reports that solvent polarity strongly influences the recovery of bioactive metabolites [60]. Isolates Dib-3, Dib-4, and Sac-12 consistently displayed the strongest activity, recording some of the lowest MIC (0.039 mg/mL) and MBC (0.156 mg/mL) values, particularly against Gram-positive bacteria (*S. aureus* and *E. faecium*) as well as the Gram-negative test pathogen *P. aeruginosa*. The MIC and MBC results of the study further reveal that the ethyl acetate and methanol extracts are effective antibacterial agents individually, but they are even more effective when acting synergistically. The majority of active extracts exhibited MBC/MIC ratios ≤ 4, indicating predominantly bactericidal rather than bacteriostatic effects.

With the exception of *Pseudomonas aeruginosa,* the bacterial response to extracts was consistently greater in Gram-positive bacteria compared to Gram-negative counterparts, in line with the previous studies that reported Gram-positive bacteria were more susceptible to fungal extract than Gram-negative bacteria [61]. This condition was presumably caused by the difference in cell wall structure of these two classes of bacteria that serves as a permeability barrier to impede the toxic substances penetration into the cells. Theoretically, the cell wall of Gram-positive bacteria consists of mainly thick and porous peptidoglycan layer without the existing of outer membrane which causes it to become permeable to most antibiotics. In contrary, the cell wall of Gram-negative bacteria contains more complex substances with outer membrane layer that caused them to be more resistant to the antibacterial agent [62].

The exception observed with *P. aeruginosa* may be explained by its unique outer membrane physiology and adaptive responses, demonstrating the exceptional potency of marine fungal metabolites. Although *P. aeruginosa* is generally considered highly resistant, certain fungal-derived secondary metabolites are known to target membrane integrity or quorum sensing pathways, which could compromise its defenses and render it more susceptible than other Gram-negative bacteria [63]. More strikingly, majority of the extracts successfully inhibited the growth of multidrug-resistant ESKAPE pathogens, where several commercial antibiotic discs (positive controls) failed. This unique observation underscores the therapeutic potential of marine fungal metabolites as alternative leads against the most clinically challenging bacterial infections. The ability of these extracts to outperform conventional antibiotics strongly suggests the presence of novel or less-exploited modes of antibacterial action, a feature of great significance in the global fight against antimicrobial resistance.

Previous studies confirmed that *Curvularia* spp. are producers of source of various biological activities and biotechnological applications [64]. Isolate Dib-3 *(Curvularia* spp*.)* exhibited strong antibacterial activity, particularly against *S. aureus* and *E. faecium*. Similarly, isolate Dib-4 (*Alternaria* sp.) was the most potent overall, with low MIC values against *P. aeruginosa*, *S. aureus,* and *E. faecium*. This is consistent with earlier reports on *A. alternata* strains producing secondary metabolites actively inhibiting the growth of drug-resistant bacteria [65]. The very low MIC/MBC values observed here highlight *A. alternata* as an outstanding candidate for further chemical exploration. Isolate Sac-12 (*Penicillium citrinum)* also demonstrated consistently strong antibacterial potential, especially against *P. aeruginosa.* This is in agreement with the well-documented antimicrobial potential of *Penicillium* species, which are known to produce distinctive metabolites showing strong inhibitory activities against drug-resistant bacteria [48,56].

The mechanism underlying these activities was supported by scanning electron microscopy (SEM) analysis, which revealed pronounced morphological alterations in bacterial cells exposed to the extracts. As shown in (Figs 10-12), *S. aureus* cells exhibited surface collapse and deformation, while *E. cloacae* displayed membrane blebbing, rupture, and cytoplasmic leakage. These ultrastructural changes suggest that the fungal metabolites act primarily through membrane-targeting mechanisms. In this case, the bioactive molecules contained in ethyl acetate and methanol (combined) extract of Dib-3, Dib-4, and Sac-12 isolates can bind strongly to the bacterial outer membrane causing the permeation of antibacterial agent across the membrane and caused the cells to become unstable and collapsed structurally that resulted to rapid cell death. This is consistent with the finding of Chatterjee and his colleagues that reported the ethyl acetate extract of *Alternaria alternata* AE1 caused the destruction of the bacterial cell wall which led to the total damage of the cell [65], as well as with general models of membrane-active natural products [66,67].

To understand the chemical constituents underlying the structural change observed by scanning electron microscopy (SEM) analysis, the ethyl acetate and methanolic extracts of the three most active fungal isolates (Dib-3, Dib-4, and Sac-12) were further subjected to GC-MS analysis. This profiling revealed detailed chromatographic information, including retention times (R_T_), relative peak areas, molecular weights, and molecular formulas (MF) of the detected compounds, as indicated in the total ion chromatograms (TICs) and summarized in Tables 10-15. The pronounced antibacterial potential of these extracts can be directly correlated with their chemically diverse profiles detected by GC-MS analysis. The extracts were dominated by fatty acids, phenolic derivatives, polyols, esterified derivatives, hydrocarbons, and nitrogen-containing compounds.

Specifically, the GC-MS profile of Dib-3 ethyl acetate extract demonstrated a high presence of phenolic compounds, fatty acid derivatives, long-chain hydrocarbons, and esters, suggesting strong membrane-active mechanisms of antimicrobial potential. Phenolic compounds such as benzeneethanol, 4-hydroxy- (commonly known as tyrosol), phenylethyl alcohol, and 2,4-di-tert-butylphenol are widely known for their antibacterial effects by disrupting bacterial membranes, inducing oxidative stress, and inhibiting essential enzymes, whereas long-chain fatty acids and esters destabilize lipid bilayers, leading to leakage of intracellular components [52,68]. Similar metabolite classes have been reported in marine and fungal-derived extracts exhibiting potent activity against MDR *Staphylococcus aureus*, *Klebsiella pneumoniae*, and *Pseudomonas aeruginosa*, supporting the antibacterial profile observed in the present study [69,70]. The preponderance of these metabolites in the ethyl acetate extract most likely explains its enhanced antibacterial performance compared to the methanolic counterpart. Likewise, the methanolic extract of Dib-3 was enriched with nucleobase derivatives, polyols, fatty acid esters, and unsaturated fatty acids, reflecting a chemically complex but relatively more polar composition. In this extract, the most abundant metabolite, 6-Azathymine, bis(methyl) ether (nucleobase derivatives) has been reported to exert potent antibacterial activities by functioning as a competitive inhibitor of bacterial growth and effectively suppressing biofilm formation in ESKAPE organisms [71,72]. Ascorbic acid derivatives (l-(+)-Ascorbic acid 2,6-dihexadecanoate) also contribute through reactive oxygen species (ROS)-mediated oxidative damage and enzymatic inhibition, amplifying antibacterial efficacy synergistically [73]. Polyols like D-mannitol are known to cause osmotic stress and membrane destabilization, while fatty acid esters enhance membrane permeability and metabolic interference [74].

The GC-MS result of ethyl acetate Dib-4 extract showed the predominance of aliphatic hydrocarbons, long-chain fatty acids, alcohols, and phenolic compounds, with cis-10-heptadecenoic acid, pentadecanoic acid, and linolelaidic acid (omega-6 trans fatty acid) as major compounds. These fatty acids are particularly effective in disrupting bacterial membranes, increasing permeability, and inducing cytoplasmic leakage, in agreement with the pronounced cellular deformation and membrane collapse evident in SEM micrographs. Similar fatty acid-rich extracts from fungal and marine origins have shown strong inhibitory activity against ESKAPE microorganisms, corroborating the strong antibacterial performance of Dib-4 observed in this study [75]. Phenolic compounds and long-chain alcohols also synergistically enhance the efficacy through oxidative and membrane-targeted mechanisms. Similarly, the methanolic extract of Dib-4 was characterized by the presence of fatty acids, sugar alcohols, ester derivatives, and nitrogen-containing heterocycles, indicating a multi-target antibacterial mode of action. Nitrogenous heterocycles, including pyrrolo-pyrazine derivatives, have been reported to interfere with DNA synthesis, enzymatic pathways, and metabolic regulation, while sugar alcohols enhance osmotic imbalance, collectively leading to bacterial death [76]. Similar chemical compositions have been observed in fungal extracts exhibiting broad-spectrum antibacterial efficacy against multidrug-resistant bacteria [77].

The GC-MS profile of the Sac-12 ethyl acetate extract revealed a dominance of long-chain fatty acids, aliphatic hydrocarbons, aromatic alcohols, and heterocyclic compounds, suggesting a strong membrane-disruptive activity. Aromatic alcohols such as phenylethyl alcohol and tryptophol are well documented for their membrane solubilizing and enzyme-inhibitory effects [78], while the long-chain fatty acids enhance membrane permeability, leading to rapid bactericidal action [75]. Comparable metabolite profiles have been reported in marine fungal extracts with potent antimicrobial properties against multi drug-resistant Gram-positive and Gram-negative bacterial pathogens [79]. Furthermore, the methanolic extract of Sac-12 displayed a high abundance of fatty acids, polyols, and esterified derivatives, reinforcing its chemically diverse and bioactive nature. Polyols and esterified fatty acids have been widely reported to exhibit synergistic antimicrobial effects by inducing osmotic imbalance, oxidative stress, and membrane destabilization, supporting the severe morphological alterations detected by SEM [80].

Overall, the integrated GC-MS profiling and SEM observations strongly indicate that the antibacterial efficacy of Dib-3, Dib-4, and Sac-12 extracts is mediated through membrane disruption, metabolite interference, oxidative stress induction, and multi-target synergistic mechanisms. Moreover, to the best of our knowledge, this is the first comprehensive report on such activity from this ecological niche. By highlighting both the taxonomic diversity and the remarkable bioactivities of these isolates, the present study validates the therapeutic potential of these extracts as promising sources of novel antimicrobial compounds for combating multi drug-resistant bacterial infections.

## Conclusions and recommendations

The study provides the first comprehensive report on fungal epiphytes associated with marine brown algae from the Kenyan coast, revealing their remarkable capacity to produce bioactive metabolites with antibacterial activity. Morphological identification and ITS-based phylogenetic analyses confirmed that the isolates belong to metabolite-rich genera: *Aspergillus, Fusarium, Alternaria, Curvularia, Penicillium,* and *Talaromyces,* which are mostly recognized for their potential to produce interesting secondary metabolites. Both ethyl acetate and methanolic extracts derived from these fungi exhibited strong inhibitory effects against the tested bacterial pathogens.

Importantly, some fungal extracts were uniquely effective against multidrug-resistant ESKAPE pathogens, even in cases where conventional antibiotic discs failed, underscoring their potential as alternative leads for combating antimicrobial resistance. Scanning electron microscopy demonstrating that these metabolites act through membrane-targeting mechanisms, causing pronounced ultrastructural damage and bacterial cell death.

Generally, the findings establish marine brown algae-associated fungal epiphytes as underexplored but highly promising reservoir for next-generation antibiotic discovery. Given the growing global challenge of antimicrobial resistance, further comprehensive chemical characterization alongside detailed mode-of-action studies of the most bioactive metabolites is strongly imperative. Continued exploration of marine fungal symbionts from this region are essential to unlock their ecological and pharmaceutical potential.

## Supporting information

S1 Table antibacterial effect data of epiphytic fungal extracts against the test microorganisms. An Excel file containing four sheets: (sheet 1) zones of inhibition data (in triplicates) for ethyl acetate extracts; (sheet 2) mean ± SD of ethyl acetate extracts; (sheet 3) zones of inhibition data (in triplicates) for methanolic extracts; and (sheet 4) mean ± SD of methanolic extracts, respectively.
(XLSX)
